# Tyrosine–Peptide Analog Modulates Extracellular Vesicles miRNAs Cargo from Mesenchymal Stem/Stromal and Cancer Cells to Drive Immunoregeneration and Tumor Suppression

**DOI:** 10.3390/biom16020243

**Published:** 2026-02-03

**Authors:** Michelle B. R. G. Ley, Karina Galoian, Daniel A. Martinez, Arianna Patel, Reanna Thomas, Tressa R. Parker, Lee Friedman, Allie L. Andryski, Francis J. Hornicek, Thomas M. Best, Dimitrios Kouroupis

**Affiliations:** 1 Department of Biomedical Engineering, University of Miami, Miami, FL 33146, USA; ley.michelle@miami.edu; 2 Department of Orthopedics, Miller School of Medicine, University of Miami, Miami, FL 33136, USA; dam286@med.miami.edu (D.A.M.); ariannapatel0928@gmail.com (A.P.); rxt1611@med.miami.edu (R.T.); trp90@med.miami.edu (T.R.P.); lrf60@miami.edu (L.F.); ala186@miami.edu (A.L.A.); fjh21@med.miami.edu (F.J.H.); txb440@med.miami.edu (T.M.B.); 3 Sylvester Comprehensive Cancer Center, Miller School of Medicine, University of Miami, Miami, FL 33136, USA; 4 Diabetes Research Institute & Cell Transplant Center, Miller School of Medicine, University of Miami, Miami, FL 33136, USA

**Keywords:** extracellular vesicles, exosomes, microRNA profiling, sarcoma, stem cells, stemness modulation, TPA therapy, tumor microenvironment, drug resistance, cell-free therapeutics

## Abstract

Soft tissue sarcoma remains challenging to treat due to its heterogeneity, stemness-associated survival programs, and resistance to conventional therapies. Extracellular vesicles (EVs) mediate tumor–stroma communication, yet how stemness-targeted therapies reshape EVs-associated miRNAs networks remains unclear. This study profiled EVs miRNAs cargo from infrapatellar fat pad mesenchymal stem/stromal cells (IFP-MSCs) and sarcoma cells (SCs) under basal conditions and following treatment with a synthetic tyrosine peptide analog (TPA). EVs were isolated, characterized, and subjected to miRNAs profiling and pathway enrichment analyses. TPA induced ≥2-fold regulation of 182 miRNAs, including 49 upregulated and 24 downregulated in IFP-MSC-EVs and 86 upregulated and 23 downregulated in SC-EVs. A conserved core of 149 miRNAs (67.1%) was shared across all EVs groups. Abundant species included miR-3960 and miR-21-5p, while TPA reduced tumor-associated miRNAs such as miR-1246 (~10-fold decrease in IFP-MSC-EVs). Pathway enrichment revealed consistent targeting of cancer, MAPK, Wnt, TGF-β, and immune signaling pathways, with modest increases in mapped gene coverage following TPA treatment. In silico analysis identified distinct EVs miRNA–gene interaction profiles, with VEGFA emerging as a recurrent predicted target. These results demonstrate that stemness-targeted modulation quantitatively reprograms EVs miRNA cargo in a cell-type-dependent manner, reshaping vesicle-mediated signaling networks in sarcoma.

## 1. Introduction

Sarcomas are highly heterogeneous mesenchymal malignancies with poor clinical outcomes and limited responsiveness to conventional therapies [1]. Increasing molecular evidence suggests that several sarcoma subtypes may arise from mesenchymal stem/stromal cells (MSCs), multipotent non-hematopoietic cells capable of differentiating into adipogenic, chondrogenic, and osteogenic lineages [2,3,4]. Both MSC and tumor cell subpopulations with stem-like properties secrete diverse extracellular vesicles (EVs) enriched in proteins, lipids, and microRNAs (miRNAs) that influence tumor progression and stromal remodeling [5,6]. As a result, disruption of EVs biogenesis, release, or uptake is emerging as a promising strategy to disrupt communication networks that sustain sarcoma aggressiveness.

Stem-like tumor cells are increasingly recognized as key drivers of relapse, metastasis, immune evasion, and resistance to cytotoxic therapies in aggressive soft tissue sarcomas. Hypothalamic neuropeptide proline-rich polypeptide-1 (PRP-1) has previously been characterized as a regulator of mesenchymal cell behavior with context-dependent antiproliferative and epigenetic activity in malignant tumors [7]. Building on these findings, a synthetic tyrosine peptide analog (TPA) developed in this laboratory was shown to significantly suppress tumor growth, inhibit refractory sarcoma progression, and extend overall survival in both primary and metastatic patient-derived xenograft models [8]. Mechanistically, TPA selectively targets cancer stem cell-like populations by downregulating EZH2 and its downstream regulators, including ALDH1A1 and NANOG. Consistent with direct impairment of self-renewal programs, TPA prevents spheroid formation in self-renewal assays, underscoring its capacity to disrupt stemness maintenance pathways central to sarcoma recurrence and treatment resistance. In vivo, TPA-mediated stemness inhibition was associated with reduced sarcoma recurrence and improved overall survival, including in combination with doxorubicin, supporting the therapeutic relevance of targeting EZH2-driven stemness programs [8].

The rationale for selecting TPA as a targeted intervention is further supported by prior studies of its parent peptide, PRP-1, which demonstrated antiproliferative and epigenetic regulatory activity in sarcoma cells while exerting beneficial effects on MSC and giant cell tumor stromal cells. Interestingly, PRP-1 has also been reported to exhibit neuroprotective and anti-neurodegenerative properties in non-malignant systems, suggesting a degree of biological selectivity that distinguishes this peptide class from broadly cytotoxic or non-specific epigenetic inhibitors. Collectively, these observations highlight TPA as a mechanistically defined and translationally relevant modulator of stemness-associated pathways in sarcoma [9,10,11,12,13,14,15,16,17].

Despite growing evidence that stemness-targeted therapies can suppress tumor progression, how such interventions reshape intercellular signaling within the sarcoma microenvironment remains poorly understood. EVs are central mediators of cell–cell communication, transporting bioactive cargo that regulates self-renewal, survival signaling, immune modulation, and drug resistance [18]. Among EVs constituents, miRNAs are of particular interest due to their ability to coordinate gene networks governing stromal–tumor crosstalk, inflammation, and tissue remodeling, while also reflecting cellular state and therapeutic modulation. In mesenchymal systems, MSC-derived EVs miRNAs have been linked to wound healing and immunomodulation, underscoring the functional relevance of miRNAs cargo composition in both regenerative and pathological contexts [19,20,21].

MSCs are relevant to this biological context because they share partial phenotypic and functional features with stem-like tumor cells, have been proposed as cells of origin for select sarcoma subtypes, and contribute to the stromal niche that supports malignant growth. Their EVs, extensively investigated in regenerative medicine, provide a valuable comparator for discerning how benign and malignant stem-like mesenchymal populations encode extracellular signaling cues without implying ontological equivalence [22,23,24,25,26,27].

To investigate how TPA-driven stemness inhibition influences EVs-mediated communication, the miRNAs cargo of EVs derived from infrapatellar fat pad-derived MSCs (IFP-MSC) and from three-dimensional spheroids generated from HT1080 soft tissue sarcoma cells (SCs) was profiled under untreated and TPA-treated conditions. Spheroid culture enriches tumor cells with enhanced self-renewal capacity, survival signaling, and therapy resistance, providing a robust model to interrogate stemness-associated signaling in sarcoma [28,29]. This comparative framework enables characterization of EVs-mediated signaling across regenerative and malignant mesenchymal populations, identification of therapy-induced alterations in stemness-linked EVs cargo, and mapping of miRNAs networks associated with chemoresistance and recurrence. By integrating stemness modulation with EVs profiling, this study provides mechanistic insight into how TPA reshapes extracellular communication circuits that contribute to sarcoma progression and treatment resistance.

## 2. Materials and Methods

### 2.1. Isolation, Culture, and Expansion of IFP-MSCs and IFP-MSC-EVs

The infrapatellar fat pad (IFP) is an adipose structure located within the knee joint, situated between the patellar tendon and the anterior tibial plateau, and serves as a rich source of multipotent mesenchymal progenitors [30]. All experiments involving human cells were conducted in accordance with relevant guidelines and regulations. Human infrapatellar fat pad-derived mesenchymal stem/stromal cells (IFP-MSC) were isolated from IFP tissue obtained from de-identified, non-arthritic patients (n = 2; male 46 years old and female 44 years old) undergoing elective knee arthroscopy at the Lennar Foundation Medical Center, University of Miami. All procedures were performed under University of Miami IRB exemption as non-human research, based on the classification of the samples as discarded tissue.

Following standardized protocols [31,32,33], IFP tissue (5–10 cc) was mechanically dissected and repeatedly washed with Dulbecco’s phosphate-buffered saline (DPBS; Sigma-Aldrich, St. Louis, MO, USA), then enzymatically digested with 235 U/mL collagenase I (Worthington Industries, Columbus, OH, USA) diluted in DPBS containing 1% bovine serum albumin (Sigma) for 2 h at 37 °C with agitation. Digestion was halted by adding complete medium consisting of Dulbecco’s modified Eagle’s medium (DMEM) low glucose (1 g/L) GlutaMAX (Thermo Fisher Scientific, Waltham, MA, USA) supplemented with 10% fetal bovine serum (FBS; VWR). The resulting cell suspension was washed and seeded at a density of 1 × 10^6^ cells per 175 cm^2^ flask in Mesenchymal Stem Cell Growth Medium 2 (PromoCell), supplemented according to the manufacturer’s instructions (MSC medium). At 48 hours post-seeding, non-adherent cells were removed by rinsing with DPBS, and fresh medium was added. All MSC cultures were maintained at 37 °C in 5% (*v*/*v*) CO_2_ until reaching 80% confluence at passage 0 (P0), then subcultured at a 1:5 ratio until passage 2 (P2). Cells were detached with TrypLE™ Select enzyme 1× (Gibco, Waltham, MA, USA) and viability was assessed using 0.4% (*w*/*v*) trypan blue (Invitrogen, Carlsbad, CA, USA). At passage 3 (P3), cells from each line were seeded into six 175 cm^2^ flasks and cultured until 60% confluence, which was designated as day 1.

On day 1, flasks were washed twice with DPBS to eliminate residual serum-derived vesicles, and fresh MSC-EVs-depleted medium was added. MSC-EVs-depleted medium was prepared by ultracentrifugation of the supplement at 120,000× *g* for 4 h at 4 °C in a Beckman Coulter Optima XPN ultracentrifuge equipped with an SW 40 Ti swinging-bucket rotor, following established protocol [34].

To evaluate the effects of TPA, two groups were established: an untreated control and a TPA-treated group. Accordingly, from days 1–3, half of the flasks received daily TPA supplementation (40 μg/mL). On day 4, conditioned medium (secretome) was collected, and cells were harvested as previously described. Secretome from all flasks was pooled and supplemented with protease inhibitor (Thermo Fisher Scientific; 4 µL per 10 mL of secretome) and immediately centrifuged at 910× *g* for 10 min at 4 °C to remove cellular debris and large vesicles. The resulting supernatant was subjected to ultracentrifugation under established conditions, and the EVs-containing pellet was resuspended in DPBS and passed through a 0.22 µm filter to ensure sterility. The final preparations were designated as IFP-MSC-EVs (untreated) and IFP-MSC-TPA-EVs (TPA-treated).

### 2.2. Isolation, Culture, and Expansion of SCs and SC-EVs

Human soft tissue sarcoma HT1080 cells (SC; ATCC^®^ CCL-121™) were cultured in fetal bovine serum (FBS), depleted Eagle’s minimum essential medium (EMEM; ATCC) supplemented with 10 ng/mL basic fibroblast growth factor (bFGF; Peprotech, Cranbury, NJ, USA), 10 ng/mL epidermal growth factor (EGF; Peprotech), and 10 μL/mL N-2 supplement (Gibco). This formulation is referred to as SC medium throughout the manuscript.

SCs were cultured in T-175 flasks (Falcon) at 37 °C in a humidified atmosphere with 5% (*v*/*v*) CO_2_ until reaching ~80% confluence. Cells were then detached with 0.25% trypsin (Gibco) and counted using a hemocytometer. Two 6-well plates (Falcon), pretreated with anti-adherence rinsing solution (StemCell Technologies, Vancouver, BC, Canada), were seeded with 2 × 10^6^ cells per well in 3 mL of SC medium. To assess the effects of TPA, cultures were divided into untreated control and TPA-treated groups, with the treated group receiving 40 μg/mL TPA on day 1. On day 2, both groups were supplemented with bFGF, EGF, and N-2 at the concentrations specified in the SC medium formulation. After four days in culture, conditioned medium (secretome) was collected, and cells were harvested and counted as described above.

Under these conditions, SCs consistently formed three-dimensional spheroids with a mean diameter of ~500 µm at the time of secretome collection. Spheroid size was maintained within this range to minimize variability in hypoxic state prior to EVs isolation.

Secretome was centrifuged at 2000× *g* for 40 min at 4 °C to remove cells and debris, and the clarified supernatant was mixed with 0.5 volumes of Total Exosome Isolation Reagent (Invitrogen) according to the manufacturer’s instructions. Following overnight incubation at 4 °C, samples were centrifuged at 10,000× *g* for 1 h at 4 °C, and the resulting pellets were resuspended in 300 μL of exosome resuspension buffer (Invitrogen). Final preparations were designated as SC-EVs (untreated) and SC-TPA-EVs (TPA-treated).

### 2.3. Nanoparticle Tracking Analysis

EVs size distribution and concentration were determined using a NanoSight NS300 system (Malvern Panalytical, Worcestershire, UK) with nanoparticle tracking analysis (NTA) software v3.4. EVs suspensions were diluted 1:10 in DPBS to achieve particle concentrations within the optimal measurement range specified by the manufacturer and analyzed at room temperature. For each sample, five 15 s videos were acquired under constant flow (approximately 50 µL/min) with fixed camera settings. Data were processed using the manufacturer’s software with a detection threshold of 5, and results are reported as particle concentration (particles/mL) and size distribution metrics (mean, D10, D50, D90).

### 2.4. Electron Microscopy Imaging

EVs morphology was assessed by transmission electron microscopy (TEM). Samples were adsorbed on carbon-coated copper grids, fixed with 2% glutaraldehyde, and stained with 2% uranyl acetate. Imaging was carried out at 80 kV using a JEOL JEM-1400 microscope, and representative micrographs were acquired with an AMT BioSprint 12 digital camera (JEOL USA, INC., MA, USA).

### 2.5. miRNAs Profile of EVs

The molecular cargo of the four EVs groups (IFP-MSC-EVs, IFP-MSC-TPA-EVs, SC-EVs, and SC-TPA-EVs) was analyzed using a curated miRNAs quantitative PCR (qPCR) array (GeneCopoeia, Rockville, MD, USA). Total miRNAs was extracted from EVs preparations using the Total Exosome RNA and Protein Isolation Kit (Thermo Fisher Scientific) according to the manufacturer’s instructions. miRNAs concentration and purity were assessed on a NanoDrop ND-1000 spectrophotometer (Thermo Fisher Scientific) using 2 µL per measurement.

Complementary DNA (cDNA) synthesis was performed by reverse-transcribing 100 ng of total EVs miRNAs with the All-in-One miRNAs First-Strand cDNA Synthesis Kit (GeneCopoeia), following the miProfile™ miRNAs PCR Arrays user manual. qPCR was carried out using pre-designed 228-miProfile human cancer exosome miRNAs qPCR arrays (QM050, GeneCopoeia, Appendix A Table A1), with 1000 ng of cDNA per sample. Reactions were run on a StepOnePlus Real-Time PCR System (Applied Biosystems) using SYBR Green detection chemistry and the manufacturer-recommended cycling parameters. Each plate included technical controls and housekeeping genes (HKGs).

Ct values were exported and processed using the GeneCopoeia online qPCR Data Analysis System (https://www.genecopoeia.com/product/qpcr/analyse/, accessed on 10 September 2025), which incorporates internal technical controls and replicates handling according to the manufacturer’s pipeline. Ct values ≥ 34 or undetermined were excluded prior to analysis, corresponding to low-abundance miRNAs near the detection limit of the assay.

Relative miRNA expression was calculated using the 2^−ΔCt^ method, with normalization to the most stable HKG across all EVs samples. SNORD48 was selected based on minimal Ct variability across experimental groups and conditions. All analyses were performed using biological duplicates.

### 2.6. Network and Pathway Analysis of EVs

The functional relevance of most abundant miRNAs was explored using the miRNet 2.0 platform (https://www.mirnet.ca, accessed on 10 October 2025), configured for ‘homo sapiens’ and ‘exosomes’ as the tissue source to reflect the biological context of EVs-mediated delivery. Experimentally validated miRNAs target interactions were obtained from miRTarBase v9.0. Interaction networks were subsequently refined by applying a betweenness centrality threshold (≥2.0) to highlight key regulatory hubs with high connectivity, following established network analysis principles [35].

Functional enrichment of target genes was assessed across multiple databases, including the Kyoto Encyclopedia of Genes and Genomes (KEGG), Reactome, and the Gene Ontology: Biological Process (Molecular Functions). KEGG and Reactome were used to map signaling, metabolic, and survival pathways, while Molecular Functions annotations provided insights into enriched cellular processes. Significance testing was performed using a hypergeometric test, and *p*-values were adjusted for multiple comparisons via the Benjamini–Hochberg false discovery rate (FDR) method. All analyses were conducted using the miRNet version current as of 15 August 2025 (maintained by the Xia Lab, McGill University).

### 2.7. In Silico Analysis of EVs Effects on Macrophages Polarization

Macrophages can differentiate into distinct activation states, including pro-inflammatory M1 and anti-inflammatory M2. The functional association between IFP-MSC-EVs and SC-EVs-derived miRNAs and M2 macrophage-related genes was assessed using the miRDB database (http://miRdb.org; accessed on 21 August 2025). Predicted interactions were based on MiRTarget scores, which range from 0 to 100. For this study, genes with prediction scores ≥ 50 were considered potential miRNAs targets [36].

### 2.8. Statistical Analysis

Statistical comparisons were performed using t-tests, where indicated, with significance defined as *p* < 0.05. In the presence of a non-normal distribution of the data, a one-way ANOVA was used for multiple comparisons. All results are presented as mean ± standard deviation and are based on independent biological samples derived from a minimum of two biological donors. All tests were performed with GraphPad Prism v10.4.2 (GraphPad Software, San Diego, CA, USA).

Given the limited number of biological replicates, statistical analyses were used to support descriptive comparisons rather than population-level inference, and results are interpreted at the pattern, fold-change, and pathway-enrichment level. Accordingly, findings are presented as exploratory and hypothesis-generating.

## 3. Results

### 3.1. Size Distributions and Particle Concentrations of IFP-MSC-EVs and SC-EVs with and Without TPA Treatment

**Figure 1:**
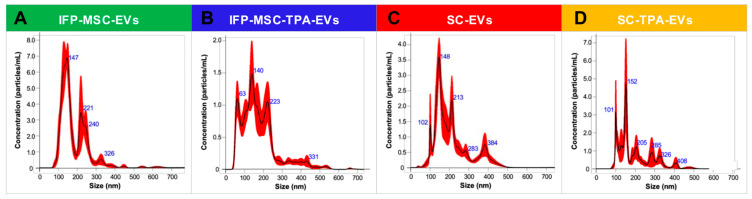
Nanoparticle tracking analysis of EVs preparations. (**A**) IFP-MSC-EVs (green) exhibited a mean size of 184.5 ± 8.5 nm with an average particle concentration of 6.11 × 10^9^ particles/mL. (**B**) IFP-MSC-TPA-EVs (blue) showed a mean size of 177.9 ± 3.4 nm with a concentration of 2.15 × 10^10^ particles/mL. (**C**) SC-EVs (red) displayed a mean size of 209.7 ± 6.7 nm and a concentration of 3.23 × 10^9^ particles/mL. (**D**) SC-TPA-EVs (yellow) had a mean size of 207.5 ± 18.3 nm with a concentration of 2.65 × 10^9^ particles/mL. These distributions confirm enrichment of vesicles within the expected size range.

Nanoparticle tracking analysis (NTA) verified the presence of EVs in all four experimental groups, revealing distinct size distributions and particle concentrations (Figure 1). IFP-MSC-EVs displayed a mean size of 184.5 ± 8.5 nm with D10, D50, and D90 values of 115.8 ± 4.3 nm, 165.4 ± 11.9 nm, and 285.9 ± 24.9 nm, respectively, and an average particle concentration of 6.11 × 10^9^ particles/mL. IFP-MSC-TPA-EVs showed a mean size of 177.9 ± 3.4 nm (D10 = 74.6 ± 5.4 nm; D50 = 156.9 ± 4.5 nm; D90 = 303.2 ± 22.9 nm) with a concentration of 2.15 × 10^10^ particles/mL. SC-EVs exhibited a mean size of 209.7 ± 6.7 nm (D10 = 123.1 ± 5.7 nm; D50 = 189.2 ± 8.3 nm; D90 = 349.0 ± 23.5 nm) and a concentration of 3.23 × 10^9^ particles/mL. SC-TPA-EVs presented a mean size of 207.5 ± 18.3 nm (D10 = 107.4 ± 3.9 nm; D50 = 174.1 ± 14.8 nm; D90 = 359.1 ± 85.8 nm) with a concentration of 2.65 × 10^9^ particles/mL.

Across all conditions, the D10–D90 span indicates heterogeneous populations encompassing small-to-medium-sized EVs. Interestingly, TPA treatment of IFP-MSCs was associated with a marked increase in EVs particle yield without substantially altering the median or upper-range particle sizes, suggesting that TPA primarily enhances vesicle release rather than shifting vesicle biogenesis toward a distinct subpopulation. Conversely, SCs produced EVs of consistently larger diameter, reflecting intrinsic differences in parental cell type and tumor origin. These findings confirm successful isolation of well-defined EVs preparations with characteristic size distributions and highlight the impact of both cell source and TPA priming on vesicle production.

### 3.2. Morphological Characterization of IFP-MSC- and SC-EVs with and Without TPA Treatment

**Figure 2:**
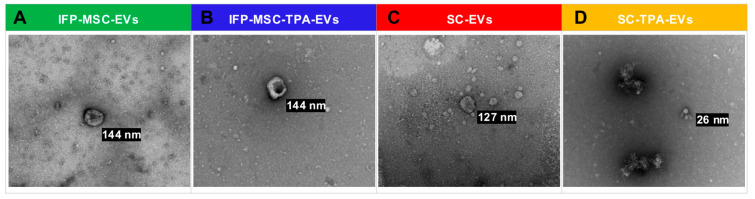
Transmission electron microscopy of EVs preparations. (**A**) IFP-MSC-EVs (green), (**B**) IFP-MSC-TPA-EVs (blue), and (**C**) SC-EVs (red) exhibit intact lipid bilayer membranes, uniform morphology, and minimal aggregation, confirming successful isolation and preservation. (**D**) SC-TPA-EVs (yellow) display irregular morphology with evidence of structural alterations, indicating treatment-dependent effects on vesicle integrity.

Transmission electron microscopy (TEM) confirmed the characteristic morphology of EVs preparations (Figure 2). IFP-MSC-EVs and IFP-MSC-TPA-EVs appeared as round to slightly ovoid particles with intact lipid bilayers, with no evidence of cellular debris or protein aggregates. SC-EVs exhibited a similar morphology, displaying well-preserved, membrane-bound vesicles consistent with small-to-medium-sized EVs. As a limitation of the present study, protein EVs marker analysis (e.g., CD9, CD63, TSG101) and negative markers assessment (e.g., calnexin) were not performed to further confirm EVs identity.

In contrast, SC-TPA–EVs displayed pronounced morphological alterations, with many vesicles appearing irregular or deformed. This phenotype is consistent with the known biological activity of TPA, which disrupts stemness-associated programs in sarcoma cells. EVs derived from stem-like tumor populations typically carry oncogenic proteins, miRNAs, and other bioactive factors that promote drug resistance, angiogenesis, and tumor progression. By impairing these stemness pathways, TPA may alter vesicle biogenesis, leading to structural changes that reflect shifts in cargo loading, membrane organization, or release dynamics.

The altered morphology observed in SC-TPA-EVs therefore suggests that TPA exerts cell-type-specific effects on EVs production, influencing both structural and potentially functional properties. Interestingly, such morphological changes were not observed in IFP-MSC-TPA-EVs, which retained the characteristic spherical morphology. These findings underscore the importance of considering both cell origin and pharmacological modulation in EVs research and highlight the utility of morphological assessment as a complementary measure of EVs identity and quality.

### 3.3. miRNAs Profiling of IFP-MSC- and SC-EVs with and Without TPA Treatment

**Figure 3:**
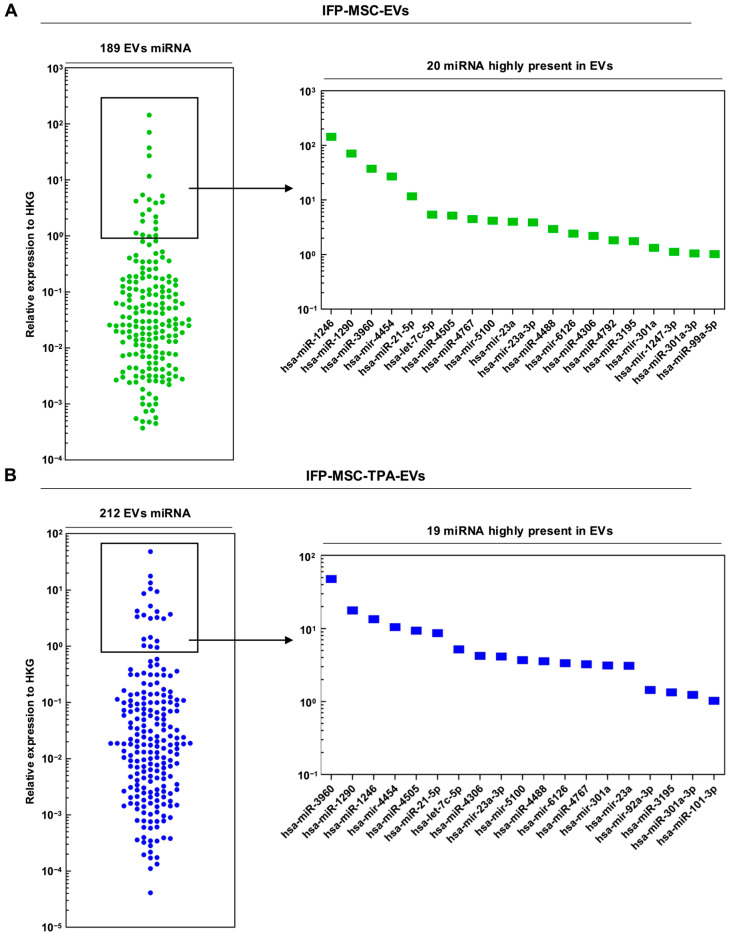

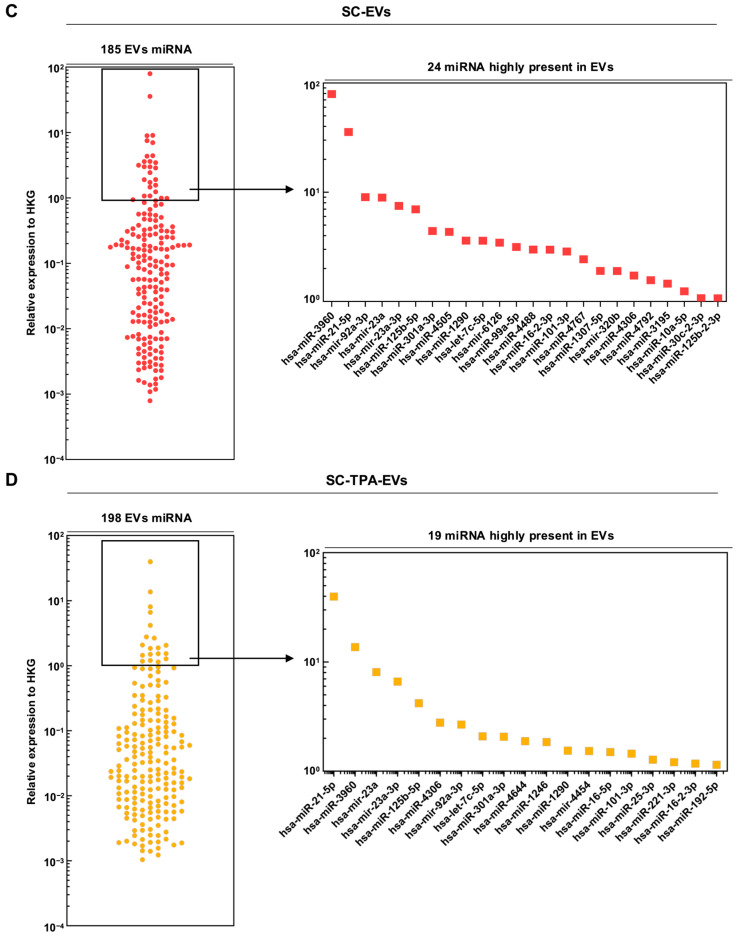
miRNAs expression profiles of EVs from different cell sources with and without TPA treatment. (**A**) IFP-MSC-EVs (green), (**B**) IFP-MSC-TPA-EVs (blue), (**C**) SC-EVs (red), and (**D**) SC-TPA-EVs (yellow). Each panel includes a swarm plot depicting ΔCt values for all detected miRNAs and a bar graph of the most abundant species. Highly expressed miRNAs (greater than 1-fold relative to HGK) are highlighted, with miR-3960 consistently ranking among the most abundant across all EVs types. Comparative profiling shows both conserved and condition-specific miRNAs signatures, with TPA treatment altering the abundance distribution in MSC- and SC-derived EVs. Data represents biological duplicates.

qPCR profiling of IFP-MSC-EVs identified 189 miRNAs, of which 20 were classified as highly expressed (Figure 3A). The top five, based on a more than 10-fold expression threshold, were miR-1246 (142.99), miR-1290 (70.72), miR-3960 (37.35), miR-4454 (26.86), and miR-21-5p (11.65). In IFP-MSC-TPA-EVs, 212 miRNAs were detected, with 19 highly expressed (Figure 3B). The top four—miR-3960 (47.79), miR-1290 (17.71), miR-1246 (13.43), and miR-4454 (10.49)—were also among the most abundant in IFP-MSC-EVs controls. Interestingly, miR-1246 showed an approximately 10-fold reduction in the TPA-treated group, whereas miR-3960 was moderately increased. For SC-EVs, 185 miRNAs were identified, with 24 highly expressed (Figure 3C). The two most abundant were miR-3960 (79.16) and miR-21-5p (35.59), both also highly expressed in IFP-MSC-EVs controls. In SC-TPA-EVs, 198 miRNAs were detected, with 19 highly expressed (Figure 3D). The top two—miR-21-5p (39.76) and miR-3960 (13.70)—were shared across all control groups. Comparative analysis revealed that miR-3960 was consistently among the most abundant across all four EVs samples, although its expression varied significantly between conditions. Raw Ct values for selected miRNAs are provided in Appendix A Table A2 and Table A3.

Among the top-expressed miRNAs in each EVs group, several were detected at moderate levels, including let-7c-5p, miR-4505, miR-4306, and miR-23a-3p, which were recurrently present in both IFP-MSC- and SC-EVs. Several low-abundance miRNAs, such as miR-4488, miR-4767, and miR-3195, were also consistently detected across multiple EVs groups. TPA treatment of SCs reduced the diversity of moderately expressed miRNAs, retaining only miR-23a, miR-23a-3p, and miR-125b-5p in this range, while shifting species that were highly abundant in SC controls, such as miR-1246 and miR-1290, into the low-expression range, indicating a redistribution in EVs miRNAs abundance. miR-23a and miR-23a-3p were detected in nearly all groups, regardless of abundance category.

The collective miRNAs expression profiles observed across IFP-MSC- and SC-derived EVs, with and without TPA treatment, demonstrate distinct yet partially overlapping repertoires, characterized by consistent representation of selective miRNAs alongside condition-specific shifts in abundance. The recurrence of certain species across all groups, together with treatment-induced redistribution of others, supports the presence of a selective miRNAs loading process that is modulated by both cell origin and external stimulation. These expression patterns form the basis for the subsequent network and pathway analyses, aimed at elucidating the broader functional implications of the identified miRNAs cargo.

### 3.4. Network and Pathway Analysis of EVs-Associated miRNAs from IFP-MSCs and SCs with and Without TPA Treatment

**Figure 4:**
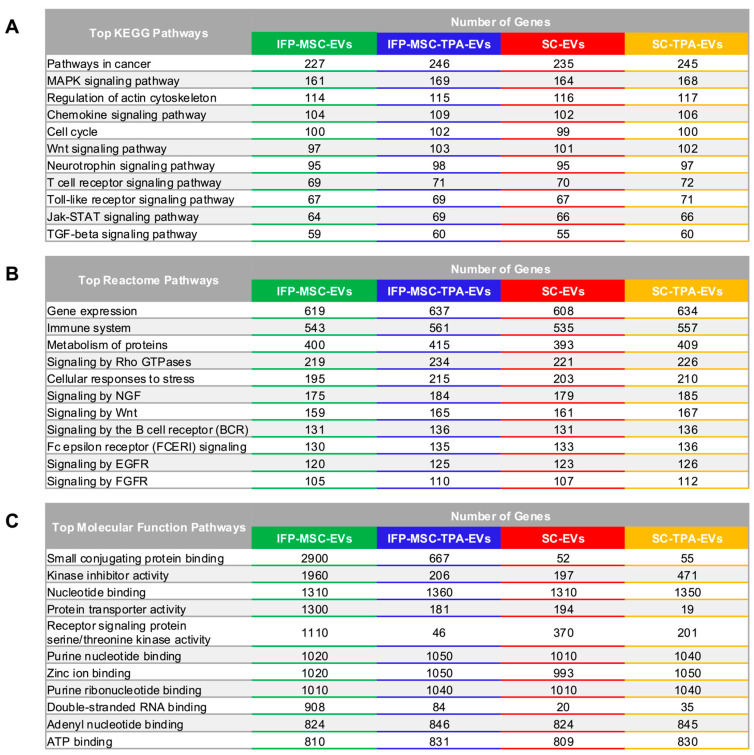
Comparative network and pathway enrichment analysis of EVs-miRNAs targets. EVs groups are color-coded as follows: IFP-MSC-EVs (green), IFP-MSC-TPA-EVs (blue), SC-EVs (red), and SC-TPA-EVs (yellow). (**A**) KEGG pathways. (**B**) Reactome pathways. (**C**) Molecular Function pathways. Overall, EVs-miRNAs converged on a conserved regulatory framework involving proliferation, immune modulation, cytoskeletal remodeling, and stress signaling, with quantitative shifts reflecting cell source and TPA treatment.

To explore the functional relevance of the cargo, the highly present miRNAs were analyzed in miRNet 2.0 using validated target gene interactions from miRTarBase v9.0. The resulting interaction map revealed a compact, hub-dense network rather than a set of isolated miRNAs target pairs, indicating that multiple miRNAs converge on common target nodes and that a limited subset of genes occupies key regulatory positions within the network. Enrichment analysis showed that the most significantly represented pathways were consistent across all four EVs groups in the KEGG, Reactome, and Molecular Function datasets. The complete lists of enriched pathways, including the number of mapped genes and corresponding *p*-values, are presented in Figure 4.

Across all four EVs groups, KEGG enrichment revealed a consistent set of core pathways. These included pathways in cancer, MAPK signaling, regulation of actin cytoskeleton, cell cycle, Wnt signaling, neurotrophin signaling, T-cell receptor signaling, toll-like receptor signaling, Jak–STAT signaling, and TGF-β signaling. Each pathway was represented by a high number of mapped target genes, indicating broad regulatory coverage. Immune-related pathways (T-cell receptor, toll-like receptor, chemokine signaling) were enriched alongside modules linked to proliferation and structural remodeling, such as regulation of actin cytoskeleton and neurotrophin signaling. While the pathway composition was qualitatively similar across all groups, TPA-treated EVs showed modest increases in mapped gene numbers in most categories, including pathways in cancer (IFP-MSC-EVs: 227 vs. IFP-MSC-TPA-EVs: 246; SC-EVs: 235 vs. SC-TPA-EVs: 245), MAPK (161 vs. 169; 164 vs. 168), and Wnt signaling (97 vs. 103; 101 vs. 102), as well as immune-related pathways such as T-cell receptor and toll-like receptor signaling. MSC-derived EVs consistently displayed slightly lower mapped gene counts compared to SC-EVs across these pathways; however, both retained strong representation across proliferation, survival, cytoskeletal, and immune signaling networks.

The Reactome outputs uncovered a consistent set of high-level functional categories across all EVs types. The largest mapped gene sets were associated with gene expression and immune system pathways, followed by metabolism of proteins and cytoskeletal control via signaling by Rho GTPases. Stress-related modules, including cellular responses to stress and signaling by NGF, were prominent in all groups, along with developmental and growth factor signaling such as Wnt, EGFR, and FGFR pathways. Immune receptor-mediated modules, including B cell receptor and Fc epsilon receptor signaling, were consistently enriched. TPA-treated EVs from both IFP-MSC and SC sources displayed modest increases in mapped gene numbers across most categories, particularly in cellular responses to stress (IFP-MSC-EVs: 195 vs. IFP-MSC-TPA-EVs: 215; SC-EVs: 203 vs. SC-TPA-EVs: 210), immune system pathways (543 vs. 561; 535 vs. 557), and receptor-mediated signaling pathways, including NGF, Wnt, and EGFR. Although MSC-derived EVs generally exhibited slightly fewer mapped gene counts than SC-derived EVs across these pathways, the overall composition remained qualitatively conserved across conditions, indicating a shared core set of functional programs.

Molecular Function enrichment exhibited consistent representation of enzymatic and cofactor-related activities across all EVs groups. Nucleotide-associated functions were among the most represented categories in every condition, including nucleotide binding (∼1310–1360 genes), purine nucleotide and ribonucleotide binding (∼1010–1050 genes), adenyl nucleotide binding (∼824–846 genes), and ATP binding (∼809–831 genes), indicating broad potential to modulate nucleotide-dependent enzymatic processes. Zinc ion binding was also highly represented across all groups (∼993–1050 genes), suggesting targeting of zinc-dependent enzymes and transcription factors. Catalytic regulation was reflected in the enrichment of kinase inhibitor activity, which increased following TPA treatment in SC-derived EVs (197 to 471 genes), and receptor signaling protein serine/threonine kinase activity, consistent with a potential capacity to influence phosphorylation-dependent signaling cascades. Small conjugating protein binding, associated with ubiquitin and SUMO pathways, was strongly enriched in MSC-derived EVs and markedly reduced following TPA treatment (2900 to 667 genes). Double-stranded RNA binding showed strong source- and treatment-dependence, being highly enriched in IFP-MSC-EVs (908 genes) but markedly reduced in IFP-MSC-TPA-EVs (84 genes) and remaining low in SC-EVs (20 genes) and SC-TPA-EVs (35 genes). Despite these quantitative shifts in rank and magnitude, the overall Molecular Function composition remained conserved across cell sources and treatments.

The conservation of pathway composition across EVs origins indicates that a common regulatory framework underlies vesicle-mediated communication, with KEGG, Reactome, and Molecular Function analyses collectively depicting a coherent network in which EVs miRNAs converge on receptor-mediated inputs, kinase cascades, cytoskeletal regulation, transcriptional control, and stress response systems. While the overall functional pathways are preserved across cell types and treatment conditions, quantitative differences in miRNAs target coverage (particularly in stress response and immune receptor signaling) were observed, including measurable shifts following TPA treatment. Such variations may be sufficient to produce distinct phenotypic outcomes in recipient cells, underscoring the importance of integrating pathway-level findings with functional assays and network-level evaluations to determine whether these EVs cargos primarily promote repair, immune modulation, or pathological signaling in specific contexts.

### 3.5. Overlap and Specificity of EVs-miRNAs Cargo with and Without TPA Treatment

**Figure 5:**
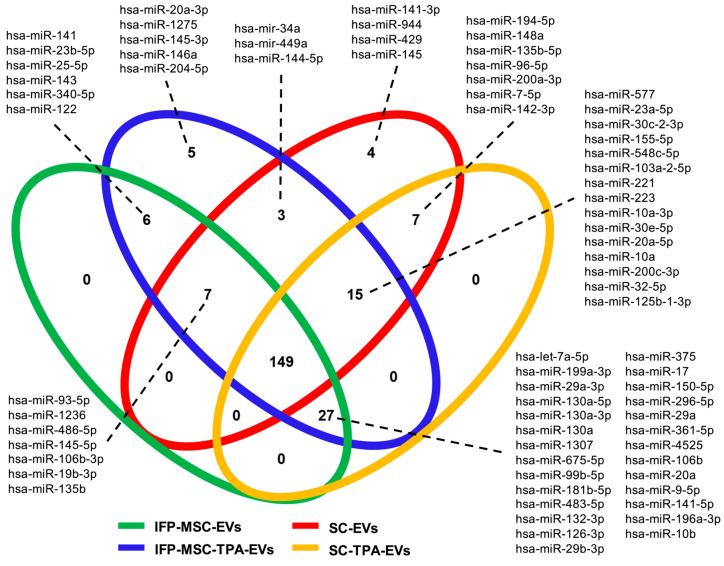
Venn diagram comparing highly expressed miRNAs in IFP-MSC-EVs (green), IFP-MSC-TPA-EVs (blue), SC-EVs (red), and SC-TPA-EVs (yellow). A total of 149 miRNAs (67.1%) were shared by all four groups, representing a conserved EVs-associated core. Group-specific subsets included 5 miRNAs (2.2%) unique to IFP-MSC-TPA-EVs and 4 miRNAs (1.8%) unique to SC-EVs, while no exclusive miRNAs were detected in IFP-MSC-EVs or SC-TPA-EVs. Pairwise overlaps ranged from 3 to 7 miRNA, and triple overlaps included 7 (3.1%), 15 (6.7%), and 27 (12.1%) miRNAs depending on group composition. Collectively, these results highlight both conserved and condition-dependent features of the EVs-miRNAs landscape. For each segment, the corresponding miRNAs are displayed adjacent to the diagram and ordered by expression level, as indicated by dashed connectors.

To evaluate the degree of conservation and divergence among IFP-MSC-EVs, IFP-MSC-TPA-EVs, SC-EVs, and SC-TPA-EVs, the top-expressed miRNAs were compared across groups using a Venn diagram (Figure 5). A total of 149 miRNAs (67.1%) were shared by all four EVs groups, representing a common core set of EVs-associated regulatory molecules. Group-specific subsets included five miRNAs (2.2%) unique to IFP-MSC-TPA-EVs and four miRNAs (1.8%) unique to SC-EVs, while no exclusive miRNAs were detected in IFP-MSC-EVs or SC-TPA-EVs.

Pairwise overlaps included six miRNAs (2.7%) shared between IFP-MSC- and IFP-MSC-TPA-EVs, seven (3.1%) between SC- and SC-TPA-EVs, and three (1.3%) between IFP-MSC-TPA-EVs and SC-EVs. No overlap was observed between IFP-MSC-EVs and SC-derived groups. Triple overlaps identified 7 miRNAs (3.1%) shared by IFP-MSC-, IFP-MSC-TPA-, and SC-EVs, 15 (6.7%) shared by IFP-MSC-TPA-, SC-, and SC-TPA-EVs, and 27 (12.1%) shared by IFP-MSC-, SC-, and SC-TPA-EVs. These results indicate the presence of a broadly conserved miRNAs core across EVs sources, with additional subsets defined by cell origin and TPA treatment.

### 3.6. TPA-Induced miRNAs Regulation

**Figure 6:**
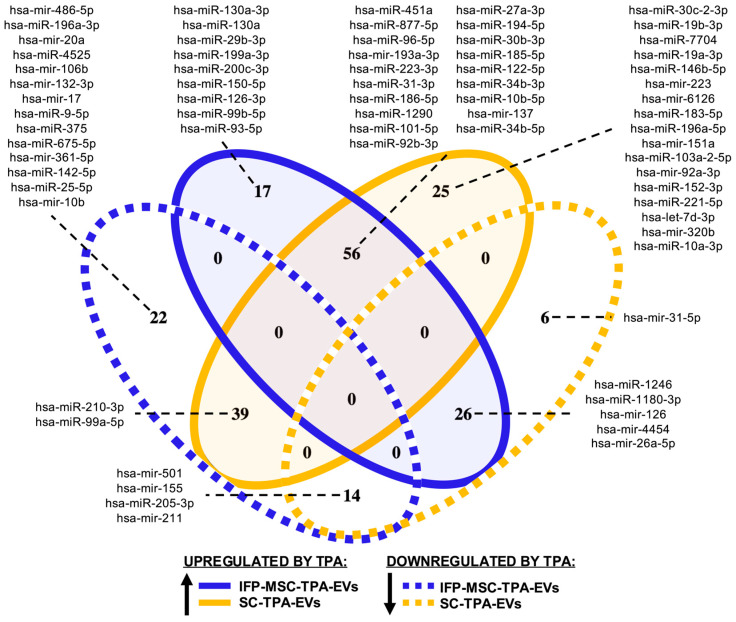
Venn diagram of miRNAs differentially expressed after TPA treatment in IFP-MSC-EVs (blue) and SC-EVs (yellow). Unique subsets included 17 upregulated in IFP-MSC, 25 upregulated in SC, 22 downregulated in IFP-MSC, and 6 downregulated in SC miRNA. Shared regions comprised 56 commonly upregulated, 14 commonly downregulated, and cross-directional overlaps of 26 upregulated in IFP-MSCs but downregulated in SCs and 39 downregulated in IFP-MSCs but upregulated in SCs, highlighting both conserved and cell-specific responses to TPA treatment. For concise presentation, only the miRNAs with the highest fold change (≥2) are listed, while the Venn diagram values represent the total number of differentially expressed miRNAs detected. For each segment, the corresponding miRNAs are displayed adjacent to the diagram and ordered by expression level, as indicated by dashed connectors.

To evaluate the effect of TPA on miRNAs regulation across cell types, up- and downregulated miRNAs were compared between IFP-MSC-TPA-EVs and SC-TPA-EVs using a Venn diagram (Figure 6). A total of 17 miRNAs (8.3%) were exclusively upregulated in IFP-MSC-TPA-EVs, while 25 miRNAs (12.2%) were uniquely upregulated in SC-TPA-EVs. In addition to these upregulated subsets, 14 miRNAs (6.8%) were downregulated in both EVs groups, whereas 22 (10.7%) and 6 (2.9%) miRNAs were exclusively downregulated in IFP-MSC-TPA-EVs and SC-TPA-EVs, respectively. The largest overlap comprised 56 miRNAs (27.3%) commonly upregulated in both groups, indicating a shared TPA-responsive miRNAs signature. Cross-directional comparisons revealed 26 miRNAs (12.7%) upregulated in IFP-MSC-TPA-EVs but downregulated in SC-TPA-EVs, and 39 miRNAs (19.0%) downregulated in IFP-MSC-TPA-EVs but upregulated in SC-TPA-EVs, highlighting divergent regulatory responses between cell types. The complete list of differentially expressed miRNAs is provided in Appendix A Table A1.

In total, 182 miRNAs displayed a fold change ≥2 in response to TPA treatment across both EVs sources; 49 miRNAs were upregulated and 24 were downregulated in IFP-MSC-TPA-EVs, whereas 86 miRNAs were upregulated and 23 were downregulated in SC-TPA-EVs. Comparative analysis revealed 19 miRNAs commonly upregulated in both groups, suggesting a partially conserved TPA-responsive signature. Cell-specific modulation was evident, with 25 miRNAs uniquely upregulated in IFP-MSC-TPA-EVs and 65 miRNAs uniquely upregulated in SC-TPA-EVs. Conversely, 18 and 14 miRNAs were exclusively downregulated in IFP-MSC- and SC-derived EVs, respectively. A smaller subset of cross-directional changes was also detected, including five miRNAs upregulated in IFP-MSC-TPA-EVs but downregulated in SC-TPA-EVs, two miRNAs showing the opposite trend, and four miRNAs commonly downregulated in both. These results highlight that while TPA induces overlapping miRNAs responses between the two EVs types, substantial cell-specific differences in magnitude and directionality underscore the distinct regulatory effects on each cell line’s secreted vesicular miRNAs content.

For clarity of visualization, Figure 6 displays only the miRNAs that were common between the full dataset of TPA-regulated miRNAs and those showing a high fold change (≥2). Instead of listing all detected elements, only representative subsets from each category are shown. These include 9 miRNAs common to all upregulated IFP-MSC miRNA, 17 shared in all upregulated SC miRNA, and 19 common upregulated miRNAs in both EVs types. Downregulated subsets comprised 14 miRNAs in IFP-MSCs, 1 in SCs, 4 shared between both, 5 upregulated in IFP-MSCs but downregulated in SCs, and 2 showing the opposite trend.

Overall, these patterns reveal both conserved and cell-specific regulatory responses to TPA treatment, indicating that protein kinase C (PKC) activation modulates EVs-associated miRNAs cargo through distinct signaling pathways in IFP-MSCs and SCs. While a partially shared set of miRNAs was upregulated in both EVs types, several subsets exhibited divergent or opposite regulatory trends, reflecting cell-type-dependent mechanisms of transcriptional and post-transcriptional control. Collectively, these findings underscore the selective and directional influence of TPA on vesicular miRNAs composition and establish the basis for subsequent analyses exploring the biological relevance and pathway associations of the most strongly modulated miRNA.

### 3.7. In Silico Prediction of EVs-Associated miRNAs–Genes Interactions in IFP-MSC-EVs and SC-EVs Linked to M2 Macrophage Polarization

**Figure 7:**
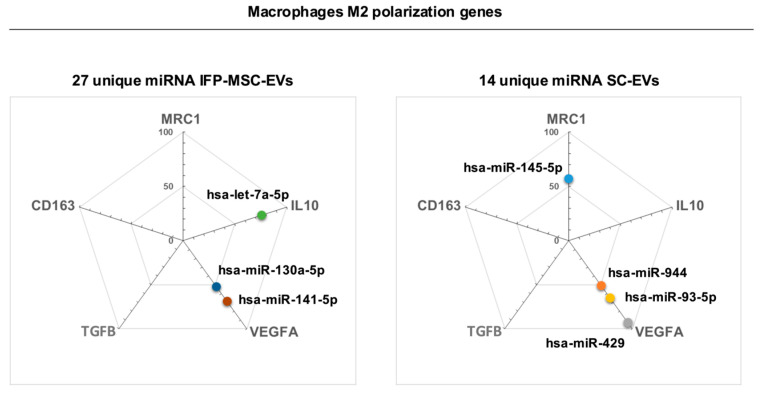
In silico analysis of IFP-MSC-EVs and SC-EVs miRNAs predicted interactions with M2 macrophage-associated genes (MRC1, CD163, IL10, TGFB, VEGFA) using the miRDB database. Candidate targets with MiRTarget scores ≥ 50% (range: 0–100%) are presented.

Target prediction analysis using miRDB revealed distinct sets of miRNAs uniquely enriched in IFP-MSC-EVs (27 miRNA) and SC-EVs (14 miRNA), with functional links to canonical M2 macrophage associated genes (MRC1, CD163, IL10, TGFB, VEGFA) (Figure 7).

Within the IFP-MSC-EVs specific set, three miRNA–gene pairs met reporting criteria and mapped to the M2 gene panel: let-7a-5p is predicted to target IL10 (75%), miR-130a-5p to VEGFA (50%), and miR-141-5p to VEGFA (60%). These interactions imply potential post-transcriptional repression of VEGFA and fine-tuning of IL10, suggesting roles in anti-inflammatory signaling and angiogenesis [37,38,39]. In the SC-EVs-specific set, four pairs mapped to the same M2 panel: miR-145-5p is predicted to target MRC1 (55%), miR-944 to VEGFA (50%), miR-93-5p to VEGFA (60%), and miR-429 to VEGFA (100%). All four miRNAs have been associated with cancer across multiple tumor types and exhibit context-dependent roles in tumor progression and therapy response [40,41,42,43,44,45]. Therefore, the SC-set indicates a tumor-related regulatory signature distinguished from the IFP-MSC profile.

VEGFA emerged as a recurrent predicted node (five unique miRNAs total: two from IFP-MSC-EVs, three from SC-EVs). In contrast, IL10 (let-7a-5p) appeared among IFP-MSC-EVs predictions, and MRC1 (miR-145-5p) among SC-EVs predictions; no unique-set predictions passed the threshold for CD163 or TGFB. Only miRNA–gene pairs with MiRTarget scores ≥ 50% are reported here. Reported percentages are miRDB probabilities (not effect sizes) and imply putative inhibitory interactions pending experimental validation.

## 4. Discussion

TPA is a synthetic analog of PRP-1. Prior studies demonstrated that PRP-1 exerts strong antiproliferative effects in chondrosarcoma through activation of tumor-suppressive programs, including modulation of tumor-suppressive miRNAs and attenuation of oncogenic regulators [9,10,11]. Mechanistically, PRP-1 functions as an inhibitor of mTOR complex 1 (mTORC1), thereby suppressing mTOR-driven proliferative signaling in mesenchymal tumor cells and contributing to its tumor-suppressive activity [12]. Consistent with this mechanism, PRP-1 displays context-dependent effects on cell proliferation, promoting expansion of bone marrow-derived MSCs while inhibiting growth of neoplastic mesenchymal cells, including giant cell tumor (GCT). This dual phenotype—supporting stromal regeneration while suppressing malignant expansion—provided the mechanistic rationale for the development of TPA as a therapeutic analog [13,14,15,16].

The antidegenerative and protective properties of TPA closely recapitulate those of PRP-1, including inhibition and disintegration of sarcoma cells and significant extension of overall survival in refractory models. The present work extends these observations by demonstrating that TPA also modulates EVs-mediated communication: TPA-induced suppression of SCs coincides with beneficial alterations in EVs miRNAs cargo, offering a mechanistic link between therapeutic stemness targeting, regenerative signaling, and intercellular transmission of anti-tumor effects [8,17].

Beyond these established intracellular mechanisms, our findings extend TPA’s dualistic biology to the EVs compartment. In MSCs, TPA did not alter the classical size distribution of secreted EVs but markedly increased vesicle yield, a pattern consistent with enhanced vesicle biogenesis. This observation is clinically meaningful: MSC-EVs are well recognized for their anti-inflammatory and pro-regenerative properties, and a pharmacologic agent that safely amplifies EVs release—without perturbing vesicle identity—could substantially strengthen cell-free regenerative therapies.

In contrast, TPA induced qualitative rather than quantitative alterations in EVs released by SCs. Particle counts and size profiles remained unchanged, yet EVs exhibited pronounced morphological remodeling, indicating a shift in vesicle composition rather than a simple suppression of vesiculation. This decoupling between EVs abundance and structure suggests that TPA reprograms SC communication by reshaping the molecular content of secreted vesicles. This reprogramming is reflected in the miRNAs landscape.

miRNAs are key post-transcriptional regulators that fine-tune gene expression by degrading target mRNA or inhibiting translation. Variations in their expression, either upregulation or downregulation, reflect adaptive cellular responses to specific stimuli and can reshape signaling networks that influence cell behavior and phenotype. Assessing miRNAs expression changes following TPA exposure offers insight into the molecular pathways modulated by this stimulus and enables the identification of miRNAs signatures linked to cellular activation, stress adaptation, and oncogenic transformation.

EVs from TPA-treated SCs were enriched for tumor-suppressive miRNAs, supporting a model in which TPA not only inhibits oncogenic signaling within SCs but redirects their paracrine outputs toward anti-tumor phenotypes. Conversely, in MSC-EVs, TPA triggered a robust upregulation of miR-451a—an 83-fold increase—consistent with its known roles in immunomodulation, inflammatory homeostasis, and lineage regulation [46]. The simultaneous induction of regenerative, anti-inflammatory EVs cargo in MSCs and tumor-suppressive cargo in SCs underscores a unifying mechanism: TPA normalizes mesenchymal communication by enhancing protective signaling while curtailing malignant messaging [47,48].

Elucidating the molecular and genetic determinants of this bifunctional response will be essential for advancing TPA toward translational applications. Our findings demonstrate that EVs-associated miRNAs represent a previously underappreciated effector arm of TPA activity, extending stemness-targeting strategies to the level of extracellular communication. These insights position EVs profiling as a powerful approach for dissecting mechanisms of drug resistance, identifying predictive biomarkers, and guiding the development of next-generation cell-free therapeutics.

For downstream interpretation, only miRNAs exhibiting >10-fold change were considered (Table 1), yielding 29 species: miR-1224-5p, miR-1246, miR-1247-3p, miR-1307-5p, miR-130b-3p, miR-1468-5p, miR-149-3p, miR-150-3p, miR-15b-5p, miR-17-3p, miR-181a-3p, miR-196a-3p, miR-19a-3p, miR-19b-3p, miR-20a, miR-23a-5p, miR-26a-5p, miR-27a-5p, miR-30c-2-3p, miR-4257, miR-451a, miR-4767, miR-486-5p, miR-574-3p, miR-675-3p, miR-7704, miR-877-5p, miR-93, and miR-96-5p. The complete dataset is provided in Appendix A Table A4.

Building on the differentially regulated species identified in Table 1, TPA selectively downregulates oncogenic miRNAs enriched in SC-EVs, including miR-26a-5p (103-fold) and miR-17-3p (39-fold)—key mediators of proliferation, survival, stemness, and metastatic signaling. Their reduced levels indicates that TPA not only inhibits SC growth but also disrupts the vesicle-mediated export of malignant cues that condition the surrounding stroma and promote immune evasion [49,50,51].

In parallel, TPA markedly upregulates tumor-suppressive miR-30c-2-3p (111-fold) and miR-181a-3p (77-fold) in SC-EVs. Both target regulatory hubs governing epithelial–mesenchymal transition (EMT), angiogenesis, and immune signaling, indicating that TPA reprograms SC-EVs toward exporting anti-tumor cues that suppress invasion and remodel the microenvironment [63,64,65,66,67].

A similar pattern emerges in the regenerative compartment. TPA downregulates miR-486-5p and miR-196a-3p (~13-fold) in IFP-MSC–EVs—changes that may appear modest numerically but are strategically consequential. These miRNAs converge on pathways governing stemness, EMT, and cancer–stroma crosstalk, meaning their reduction lowers the risk that MSC-EVs inadvertently reinforce tumor-promoting programs cancers [84,85,86,87,88,89].

Conversely, TPA strongly upregulates miR-451a (83-fold) and miR-1247-3p (11-fold) in IFP-MSC–EVs. miR-451a, in particular, occupies a central regulatory position in MSC immunomodulation, macrophage polarization, metabolic homeostasis, and inflammatory resolution. Its induction predicts enhanced regenerative and anti-inflammatory EVs outputs capable of stabilizing tissue environments and counteracting tumor-driven remodeling [91,92,93,94,95,96,97].

Together, these patterns reveal that the therapeutic impact of TPA is not determined by fold-change magnitude but by selective rewiring of miRNAs regulatory hubs across tumor and stromal EVs populations. By suppressing EVs-associated oncogenic nodes while amplifying anti-tumor and pro-regenerative mediators, TPA shifts vesicle-mediated communication toward outcomes that collectively weaken tumor-supportive pathways, limit invasive potential, and enhance reparative signaling.

To determine how these coordinated miRNAs shifts manifest at the signaling level, we next examined pathway enrichment across KEGG, Reactome, and Molecular Function analyses. EVs-associated miRNAs displayed a conserved regulatory architecture: across cell types and TPA conditions, EVs converged on receptor-driven pathways (EGFR, FGFR, T-cell, toll-like) and core intracellular cascades (MAPK, Wnt, TGF-β, Jak–STAT), extending to downstream regulators of transcription, cytoskeletal remodeling, proliferation, and stress adaptation. This consistent convergence indicates that EVs miRNAs function through an integrated regulatory scaffold rather than isolated pathways.

Although the number of enriched genes across eleven canonical KEGG/Reactome pathways remained stable between MSC- and SC-EVs, Molecular Function analysis exposed selective quantitative shifts driven by cell origin and TPA treatment. These changes involved small-conjugating protein binding, kinase inhibitor activity, protein transporter activity, serine/threonine kinase regulation, and double-stranded RNA binding—features that suggest miRNAs modulate biochemical amplitude at key molecular nodes without altering the overall signaling framework.

Enrichment of ubiquitin-like modifier binding implicates modulation of proteostasis and post-translational control, while shifts in kinase inhibitor and serine/threonine kinase regulation point to fine-tuning of MAPK and Jak–STAT activity. Changes in transporter activity indicate effects on intracellular trafficking and metabolic adaptation, and variation in double-stranded RNA (dsRNA) binding suggests potential influence on innate immune sensing. Together, these findings support a model in which EVs maintain a stable signaling core but adjust regulatory intensity to shape growth, immune, and stress-response programs in recipient cells.

Convergence on VEGFA across EVs groups underscores its role as a central regulatory hub linking angiogenesis, inflammation, and macrophage biology. In the sarcoma tumor microenvironment, tumor-associated macrophages (TAMs) frequently adopt an M2-like polarization state and contribute to immune suppression and tumor progression through mediators that include VEGF/VEGFA, IL-10, and TGF-β, alongside pro-remodeling programs that facilitate invasion and vascularization [103]. The presence of multiple miRNAs predicted to target VEGFA therefore supports the possibility of coordinated post-transcriptional buffering capable of modulating vascular and inflammatory dynamics, particularly within macrophage–EVs interactions.

By contrast, divergence among M2-associated genes indicates source-dependent biases in EVs signaling. IFP-MSC-EVs-specific predictions centered on IL10 and VEGFA, consistent with an immunoregulatory and regenerative axis characteristic of MSC-derived EVs and their capacity to modulate macrophage polarization and inflammatory tone [104]. In contrast, SC-EVs-specific predictions included MRC1 (CD206)—a hallmark of M2-like tumor-associated macrophages—indicating reprogramming toward phenotypes that sustain immune evasion and oncogenic progression.

These patterns mirror established distinctions: tumor-derived EVs drive pro-tumor macrophage polarization, whereas mesenchymal-derived EVs support controlled inflammation and tissue repair [105]. Collectively, the shared focus on VEGFA and source-specific targeting of distinct M2-associated nodes illustrates how EVs origin can impose different regulatory pressures—either constraining angiogenic and inflammatory outputs in a repair-oriented context (IFP-MSC-EVs) or favoring macrophage remodeling programs that reinforce tumor progression (SC-EVs).

Despite growing clinical interest, the translation of EVs-based therapies remains limited by unresolved regulatory and technical challenges. Inconsistent nomenclature and incomplete methodological reporting persist across studies, despite ISEV recommendations favoring classification based on measurable physical or molecular properties rather than presumed biogenesis [106,107,108,109]. At the technical level, no universally accepted isolation and characterization strategy simultaneously ensures high purity, integrity, and scalability, complicating reproducibility and clinical manufacturing [110,111]. Regulatory uncertainties are further driven by limited understanding of long-term safety, optimal dosing, biodistribution, immunogenicity, and off-target effects in humans. Although early dose-escalation clinical studies using MSC-derived EVs represent important progress, therapeutic windows remain incompletely defined [112,113]. Finally, emerging alternative administration routes introduce additional complexity due to variable EVs trafficking, cellular uptake, and intracellular retention, underscoring the need for harmonized standards and mechanistic insight to enable reliable clinical translation [114].

## 5. Conclusions

This work defines the miRNAs architecture of EVs from regenerative IFP-MSCs and malignant SCs, revealing how cell origin and TPA intervention rewire vesicle-mediated communication. 

By identifying EVs-associated miRNAs that govern key oncogenic and regenerative pathways, these findings highlight actionable regulatory nodes that may be leveraged to counter refractory, treatment-resistant cancers. However, pathway analysis suggests that potential mechanisms and functional validation (e.g., macrophage polarization assays, angiogenesis assays, tumor cell proliferation/migration assays with EV treatments) are required. Together, this study establishes EVs-miRNAs profiling as a strategic framework for developing next-generation therapies for aggressive malignancies lacking effective clinical options.

## Figures and Tables

**Table 1:** miRNAs up- and downregulated (fold change ≥ 10) between SC-TPA-EVs (yellow highlighted) and IFP-MSC-TPA-EVs (blue highlighted).

EVs Type	TPA Regulation	miRNAs	Fold-Change	References
SC-TPA-EVs	Down	miR-26a-5p	103	[49,50]
		miR-17-3p	39	[51]
		miR-150-3p	34	[52,53]
		miR-574-3p	18	[54,55]
		miR-1224-5p	17	[56]
		miR-93	14	[57,58,59]
		miR-675-3p	13	[60,61]
		miR-27a-5p	10	[62]
SC-TPA-EVs	Up	miR-30c-2-3p	111	[63,64,65]
		miR-181a-3p	77	[66,67]
		miR-19b-3p	72	[68]
		miR-23a-5p	60	[69,70]
		miR-1307-5p	40	[71]
		miR-15b-5p	29	[72,73]
		miR-4767	26	[74]
		miR-149-3p	13	[75,76]
		miR-4257	12	[77]
		miR-130b-3p	12	[78,79]
		miR-1468-5p	11	[80]
		miR-7704	11	[81]
		miR-19a-3p	10	[82,83]
IFP-MSC-TPA-EVs	Down	miR-486-5p	13	[84,85]
		miR-196a-3p	13	[86,87,88,89]
		miR-20a	11	[90]
IFP-MSC-TPA-EVs	Up	miR-451a	83	[91,92,93,94,95,96]
		miR-1247-3p	11	[97]
		miR-1246	11	[98,99]
		miR-877-5p	10	[100]
		miR-96-5p	10	[101,102]

## Data Availability

The original contributions presented in this study are included in the article. Further inquiries can be directed at the corresponding author.

## Abbreviations

The following abbreviations are used in this manuscript:

bFGF	Basic fibroblast growth factor
cDNA	Complementary DNA
DMEM	Dulbecco’s modified Eagle’s medium
DPBS	Dulbecco’s phosphate-buffered saline
EGF	Epidermal growth factor
EMEM	Eagle’s minimum essential medium
EVs	Extracellular vesicles
FBS	Fetal bovine serum
FDR	Benjamini–Hochberg false discovery rate
GCT	Giant cell tumor
HKG	Housekeeping gene
IFP-MSC	Human infrapatellar fat pad-derived mesenchymal stem/stromal cells
KEGG	Kyoto Encyclopedia of Genes and Genomes
miRNAs	MicroRNAs
MSC	Mesenchymal stem/stromal cells
NTA	Nanoparticle tracking analysis
PRP-1	Proline-rich polypeptide-1
qPCR	Quantitative PCR
SC	Sarcoma cells
TEM	Transmission electron microscopy
TPA	Tyrosine peptide analog

## Appendix A

**Table A1:** GeneCopoeia—miProfile human cancer exosome miRNA qPCR array.

Mature miRNA ID			
miR-142-5p	miR-195-3p	miR-194-5p	miR-150-5p
miR-146b-5p	miR-203a-3p	miR-215-5p	miR-17-3p
miR-223-3p	miR-203b-5p	miR-182-5p	miR-181b-5p
miR-144-3p	miR-203b-3p	miR-19a-3p	miR-26a-5p
miR-151a-5p	miR-221-5p	miR-429	miR-29b-3p
miR-25-3p	miR-221-3p	miR-142-3p	miR-4454
miR-144-5p	miR-222-5p	miR-193a	miR-574-3p
miR-16-5p	miR-20b	miR-4772-3p	miR-483-5p
miR-301a-3p	miR-21	miR-125a-3p	miR-375
miR-1468-5p	miR-193a-3p	miR-96-5p	miR-141-5p
miR-186-5p	miR-411	miR-19a-5p	miR-1275
miR-497-5p	miR-5100	miR-548c-5p	miR-574-5p
miR-146b-3p	miR-23a-3p	miR-149-5p	let-7a-5p
let-7f-5p	miR-1247-3p	miR-149-3p	miR-106b
miR-1290	miR-145	miR-486-5p	miR-17
miR-181a-2-3p	miR-221	miR-92a-3p	miR-20a
let-7g-5p	miR-222	miR-23a-5p	miR-93
miR-146a-5p	miR-223	miR-181a-5p	miR-99b-5p
miR-20a-5p	miR-34a	miR-3195	miR-1224-5p
miR-106b-3p	miR-449a	miR-3960	miR-9-5p
miR-155-5p	miR-34a-5p	miR-4792	miR-1246
miR-30e-3p	miR-34a-3p	miR-1307-5p	miR-141
miR-16-2-3p	miR-34b-5p	miR-181a-3p	miR-200c
miR-339-5p	miR-34b-3p	miR-877-5p	miR-340-5p
miR-1260b	miR-34c-5p	miR-130b-3p	miR-130a-3p
miR-17-5p	miR-34c-3p	miR-423-3p	miR-27a-5p
miR-4488	miR-1236	miR-4767	miR-191-3p
miR-23a	miR-125b-5p	miR-92b-3p	miR-29a-3p
miR-24-3p	miR-125b-1-3p	miR-15b-5p	miR-199a-3p
miR-205-5p	miR-125b-2-3p	miR-320b	miR-204-5p
miR-30e-5p	miR-224-5p	miR-135b-5p	miR-150-3p
miR-103a-3p	miR-224-3p	miR-193b-3p	miR-205-3p
miR-4505	miR-30d-5p	miR-200c-3p	miR-25-5p
miR-185-5p	let-7d-3p	miR-31-5p	miR-130a-5p
miR-4741	miR-135b	miR-455-5p	miR-23b-5p
let-7c-5p	miR-148a	miR-196a-5p	miR-217-5p
miR-15a	miR-27a	miR-32-5p	miR-1307
miR-210-3p	let-7b	miR-7-5p	miR-132-3p
miR-30b-5p	miR-18a	miR-152-3p	miR-20a-3p
miR-411-5p	miR-211	miR-200a-3p	miR-296-5p
miR-21-5p	miR-211-5p	miR-944	miR-130a
miR-451a	miR-4644	miR-1180-3p	miR-501
miR-23b-3p	miR-10a	miR-27a-3p	miR-30a-3p
miR-222-3p	miR-137	miR-31-3p	miR-361-5p
miR-30b-3p	miR-151a	miR-452-5p	miR-155
miR-21-3p	miR-10a-3p	miR-6126	miR-126
miR-10b-5p	miR-183-5p	miR-93-5p	miR-196a-3p
miR-30c-5p	miR-19b-3p	miR-101-5p	miR-301a
miR-4257	miR-577	miR-99a-5p	miR-4306
miR-30c-1-3p	miR-10a-5p	miR-93-3p	miR-146a
miR-30c-2-3p	miR-191-5p	miR-1307-3p	miR-10b
miR-103a-1-5p	miR-200a-5p	miR-101-3p	miR-4525
miR-103a-2-5p	miR-584-5p	miR-145-5p	miR-29a
miR-103b	miR-192-5p	miR-375-5p	miR-122
miR-122-5p	miR-200b-3p	miR-145-3p	miR-143
miR-122-3p	miR-7704	miR-200c-5p	miR-675-5p
miR-195-5p	miR-141-3p	miR-126-3p	miR-675-3p

**Table A2:** Raw qPCR Ct values for IFP-MSC-EVs and IFP-MSC-TPA-EVs ranked from highest to lowest.

IFP-MSC-EVs		IFP-MSC-TPA-EVs	
miRNA	Ct Value	miRNA	Ct Value
miR-1246	142.9885	miR-3960	47.7891
miR-1290	70.7171	miR-1290	17.7126
miR-3960	37.3535	miR-1246	13.4352
miR-4454	26.8637	miR-4454	10.4943
miR-21-5p	11.6459	miR-4505	9.3812
let-7c-5p	5.3680	miR-21-5p	8.6487
miR-4505	5.1712	let-7c-5p	5.2008
miR-4767	4.4596	miR-4306	4.2288
miR-5100	4.1678	miR-23a-3p	4.1506
miR-23a	3.9829	miR-5100	3.6939
miR-23a-3p	3.8816	miR-4488	3.5748
miR-4488	2.9396	miR-6126	3.3558
miR-6126	2.4150	miR-4767	3.2470
miR-4306	2.1936	miR-301a	3.1258
miR-4792	1.8253	miR-23a	3.0971
miR-3195	1.7587	miR-92a-3p	1.4409
miR-301a	1.3282	miR-3195	1.3351
miR-1247-3p	1.1214	miR-301a-3p	1.2364
miR-301a-3p	1.0447	miR-101-3p	1.0254
miR-99a-5p	1.0178	miR-4792	0.9890
miR-101-3p	0.9791	miR-7704	0.9542
miR-221-3p	0.8086	miR-155	0.5913
miR-7704	0.7940	miR-125b-5p	0.5368
miR-125b-5p	0.6934	miR-497-5p	0.4688
let-7a-5p	0.5205	miR-1224-5p	0.4420
miR-199a-3p	0.4857	miR-574-5p	0.3872
miR-1224-5p	0.4491	miR-210-3p	0.3865
miR-497-5p	0.4148	miR-29a-3p	0.3603
miR-4644	0.4027	miR-92b-3p	0.3332
miR-16-5p	0.3580	miR-4644	0.3137
miR-29a-3p	0.3565	miR-16-2-3p	0.3125
miR-574-5p	0.3472	miR-99a-5p	0.3108
miR-25-3p	0.3449	miR-320b	0.3102
miR-320b	0.3427	let-7a-5p	0.3051
miR-451a	0.2685	miR-205-3p	0.2979
miR-19a-5p	0.2600	miR-221-3p	0.2235
miR-16-2-3p	0.2503	miR-150-3p	0.2183
miR-27a-3p	0.2170	miR-130a-5p	0.2071
miR-34b-5p	0.1897	miR-25-3p	0.1709
miR-24-3p	0.1892	miR-675-5p	0.1631
let-7f-5p	0.1890	miR-125a-3p	0.1549
miR-195-5p	0.1880	miR-1260b	0.1522
miR-92b-3p	0.1779	miR-16-5p	0.1422
miR-10a-5p	0.1772	miR-675-3p	0.1412
miR-1260b	0.1711	miR-20b	0.1394
miR-20b	0.1668	miR-27a-5p	0.1366
miR-125a-3p	0.1628	let-7f-5p	0.1347
miR-26a-5p	0.1519	miR-574-3p	0.1252
miR-150-3p	0.1502	miR-15a	0.1195
miR-130a-5p	0.1494	miR-1307-3p	0.1135
miR-17-3p	0.1321	miR-4741	0.1115
miR-574-3p	0.1253	miR-192-5p	0.1085
miR-192-5p	0.1243	miR-125b-2-3p	0.1077
miR-17-5p	0.1237	miR-199a-3p	0.1027
miR-675-3p	0.1146	miR-1247-3p	0.1006
miR-193a-3p	0.1113	miR-132-3p	0.0998
miR-92a-3p	0.1094	miR-30a-3p	0.0995
miR-27a-5p	0.1050	miR-191-3p	0.0989
miR-205-3p	0.1030	miR-191-5p	0.0948
miR-155	0.1016	miR-34b-5p	0.0944
miR-30a-3p	0.0981	miR-10a-5p	0.0900
miR-4741	0.0909	miR-17-3p	0.0877
miR-23b-3p	0.0899	miR-93	0.0770
miR-210-3p	0.0892	miR-93-3p	0.0766
miR-1307-3p	0.0873	miR-23b-3p	0.0743
miR-126	0.0813	miR-26a-5p	0.0725
miR-130a-3p	0.0811	miR-501	0.0724
miR-125b-2-3p	0.0795	miR-4257	0.0705
miR-103a-3p	0.0712	miR-27a	0.0699
miR-130a	0.0682	miR-195-5p	0.0687
miR-1307	0.0627	miR-193b-3p	0.0664
miR-15a	0.0625	miR-877-5p	0.0626
miR-195-3p	0.0615	miR-211-5p	0.0609
miR-191-5p	0.0610	miR-17	0.0588
miR-151a-5p	0.0599	miR-375	0.0582
miR-193b-3p	0.0584	miR-1307	0.0518
miR-15b-5p	0.0571	miR-195-3p	0.0511
miR-191-3p	0.0527	miR-483-5p	0.0500
miR-4257	0.0522	miR-1180-3p	0.0494
miR-877-5p	0.0517	miR-30c-5p	0.0436
miR-675-5p	0.0511	miR-24-3p	0.0411
miR-10b-5p	0.0508	miR-375-5p	0.0405
miR-99b-5p	0.0452	miR-423-3p	0.0385
miR-151a	0.0433	miR-211	0.0366
miR-1307-5p	0.0425	miR-1307-5p	0.0356
miR-34a-5p	0.0421	miR-21-3p	0.0342
miR-93	0.0414	miR-20a	0.0324
miR-27a	0.0360	miR-142-5p	0.0321
miR-93-3p	0.0349	miR-196a-3p	0.0309
miR-181b-5p	0.0330	miR-103a-3p	0.0305
miR-30b-3p	0.0321	miR-21	0.0270
let-7g-5p	0.0304	miR-151a-5p	0.0253
miR-21-3p	0.0300	miR-19a-5p	0.0249
miR-181a-5p	0.0300	miR-27a-3p	0.0243
miR-1180-3p	0.0298	miR-4525	0.0240
miR-122-5p	0.0297	miR-10b-5p	0.0232
miR-1468-5p	0.0273	miR-203b-3p	0.0224
miR-193a	0.0266	miR-200a-5p	0.0222
miR-30c-5p	0.0262	miR-1468-5p	0.0211
miR-483-5p	0.0259	miR-411-5p	0.0205
miR-30e-3p	0.0256	miR-151a	0.0202
miR-21	0.0255	miR-126	0.0188
miR-222-3p	0.0250	miR-15b-5p	0.0188
miR-375-5p	0.0248	let-7g-5p	0.0187
miR-211-5p	0.0247	miR-339-5p	0.0184
miR-423-3p	0.0241	miR-181a-5p	0.0183
miR-132-3p	0.0234	miR-181b-5p	0.0183
miR-126-3p	0.0226	miR-222-5p	0.0181
miR-30b-5p	0.0225	miR-99b-5p	0.0177
miR-411-5p	0.0217	miR-17-5p	0.0176
miR-203b-3p	0.0199	miR-30b-5p	0.0174
miR-186-5p	0.0199	let-7d-3p	0.0165
miR-29b-3p	0.0197	miR-411	0.0164
miR-222	0.0191	miR-34a-3p	0.0153
miR-222-5p	0.0188	miR-106b	0.0150
miR-411	0.0186	miR-361-5p	0.0137
miR-375	0.0180	miR-30e-3p	0.0133
miR-185-5p	0.0176	miR-122-5p	0.0131
miR-200a-5p	0.0169	miR-30b-3p	0.0130
miR-17	0.0165	miR-584-5p	0.0130
miR-211	0.0164	miR-224-3p	0.0129
miR-339-5p	0.0155	miR-103a-1-5p	0.0120
miR-223-3p	0.0154	miR-193a-3p	0.0114
miR-103a-1-5p	0.0149	miR-9-5p	0.0105
miR-31-3p	0.0144	miR-130a	0.0105
miR-30d-5p	0.0140	miR-221-5p	0.0104
miR-19a-3p	0.0138	miR-130a-3p	0.0100
miR-584-5p	0.0130	miR-149-5p	0.0095
miR-34a-3p	0.0130	miR-34a-5p	0.0094
miR-142-5p	0.0127	miR-30d-5p	0.0092
let-7d-3p	0.0113	miR-103b	0.0091
miR-215-5p	0.0113	miR-222-3p	0.0083
miR-224-5p	0.0109	miR-19a-3p	0.0082
miR-34c-5p	0.0106	miR-181a-2-3p	0.0081
miR-93-5p	0.0087	miR-185-5p	0.0077
miR-501	0.0084	miR-126-3p	0.0073
miR-150-5p	0.0081	miR-34c-5p	0.0073
miR-296-5p	0.0079	let-7b	0.0073
miR-224-3p	0.0078	miR-122	0.0070
let-7b	0.0076	miR-224-5p	0.0068
miR-452-5p	0.0076	miR-146b-5p	0.0064
miR-34c-3p	0.0072	miR-196a-5p	0.0061
miR-149-3p	0.0070	miR-182-5p	0.0059
miR-181a-2-3p	0.0070	miR-31-3p	0.0055
miR-34b-3p	0.0068	miR-193a	0.0054
miR-103b	0.0065	miR-296-5p	0.0049
miR-203b-5p	0.0054	miR-222	0.0048
miR-1236	0.0053	miR-205-5p	0.0048
miR-205-5p	0.0049	miR-215-5p	0.0045
miR-29a	0.0044	miR-203b-5p	0.0045
miR-361-5p	0.0044	miR-186-5p	0.0044
miR-146a-5p	0.0043	miR-29a	0.0041
miR-30c-1-3p	0.0042	miR-486-5p	0.0039
miR-4772-3p	0.0042	miR-145-5p	0.0035
miR-130b-3p	0.0042	miR-29b-3p	0.0034
miR-486-5p	0.0038	miR-451a	0.0032
miR-4525	0.0037	miR-152-3p	0.0031
miR-106b	0.0035	miR-34b-3p	0.0030
miR-145-5p	0.0032	miR-20a-3p	0.0028
miR-152-3p	0.0031	miR-141-5p	0.0028
miR-182-5p	0.0031	miR-223-3p	0.0028
miR-31-5p	0.0031	miR-10b	0.0026
miR-20a	0.0031	miR-577	0.0026
miR-9-5p	0.0030	miR-150-5p	0.0025
miR-146b-3p	0.0030	miR-203a-3p	0.0025
miR-203a-3p	0.0028	miR-30c-1-3p	0.0024
miR-149-5p	0.0027	miR-340-5p	0.0023
miR-196a-5p	0.0027	miR-23a-5p	0.0021
miR-146b-5p	0.0026	miR-30c-2-3p	0.0021
miR-106b-3p	0.0026	miR-18a	0.0021
miR-141-5p	0.0025	miR-155-5p	0.0020
miR-221-5p	0.0024	miR-25-5p	0.0018
miR-196a-3p	0.0024	miR-149-3p	0.0018
miR-18a	0.0022	miR-141	0.0018
miR-137	0.0018	miR-548c-5p	0.0016
miR-200b-3p	0.0015	miR-137	0.0016
miR-101-5p	0.0013	miR-452-5p	0.0016
miR-10b	0.0013	miR-181a-3p	0.0015
miR-181a-3p	0.0010	miR-106b-3p	0.0015
miR-141	0.0010	miR-103a-2-5p	0.0014
miR-23b-5p	0.0010	miR-1236	0.0014
miR-25-5p	0.0008	miR-183-5p	0.0014
miR-183-5p	0.0007	miR-4772-3p	0.0014
miR-143	0.0006	miR-146b-3p	0.0013
miR-340-5p	0.0005	miR-221	0.0011
miR-19b-3p	0.0005	miR-34c-3p	0.0011
miR-135b	0.0005	miR-455-5p	0.0010
miR-122	0.0004	miR-23b-5p	0.0010
miR-455-5p	0.0004	miR-34a	0.0009
		miR-223	0.0008
		miR-10a-3p	0.0008
		miR-146a-5p	0.0008
		miR-1275	0.0008
		miR-31-5p	0.0008
		miR-101-5p	0.0006
		miR-145-3p	0.0006
		miR-30e-5p	0.0006
		miR-130b-3p	0.0004
		miR-93-5p	0.0004
		miR-449a	0.0004
		miR-200b-3p	0.0004
		miR-20a-5p	0.0003
		miR-143	0.0003
		miR-135b	0.0003
		miR-144-5p	0.0003
		miR-19b-3p	0.0002
		miR-10a	0.0002
		miR-200c-3p	0.0002
		miR-32-5p	0.0002
		miR-125b-1-3p	0.0001
		miR-146a	0.0001
		miR-204-5p	0.00004

**Table A3:** Raw qPCR Ct values for SC-EVs and SC-TPA-EVs ranked from highest to lowest.

SC-EVs		SC-TPA-EVs	
miRNA	Ct Value	miRNA	Ct Value
miR-3960	79.1582	miR-21-5p	39.7624
miR-21-5p	35.5884	miR-3960	13.7053
miR-92a-3p	9.0213	miR-23a	8.0930
miR-23a	8.9383	miR-23a-3p	6.6192
miR-23a-3p	7.4988	miR-125b-5p	4.1892
miR-125b-5p	6.9805	miR-4306	2.7830
miR-301a-3p	4.4178	miR-92a-3p	2.6697
miR-4505	4.3369	let-7c-5p	2.0849
miR-1290	3.6050	miR-301a-3p	2.0658
let-7c-5p	3.5967	miR-4644	1.8834
miR-6126	3.4581	miR-1246	1.8489
miR-99a-5p	3.1529	miR-1290	1.5404
miR-4488	2.9897	miR-4454	1.5298
miR-16-2-3p	2.9828	miR-16-5p	1.4983
miR-101-3p	2.8679	miR-101-3p	1.4473
miR-4767	2.4340	miR-25-3p	1.2775
miR-1307-5p	1.9053	miR-221-3p	1.2086
miR-320b	1.9009	miR-16-2-3p	1.1728
miR-4306	1.7291	miR-192-5p	1.1460
miR-4792	1.5655	miR-4488	0.9548
miR-3195	1.4573	miR-4505	0.9374
miR-10a-5p	1.2397	miR-301a	0.9244
miR-30c-2-3p	1.0718	miR-99a-5p	0.9013
miR-125b-2-3p	1.0693	miR-320b	0.8992
miR-4644	0.9908	miR-125b-2-3p	0.8085
miR-7704	0.9862	miR-195-5p	0.6990
miR-25-3p	0.9395	miR-29a-3p	0.5960
miR-16-5p	0.9075	miR-6126	0.5574
miR-4257	0.8467	miR-1180-3p	0.5396
miR-221-3p	0.7992	miR-24-3p	0.5070
miR-192-5p	0.7649	miR-27a	0.4954
miR-301a	0.6659	miR-10a-5p	0.4819
miR-191-5p	0.5730	miR-125a-3p	0.3495
miR-23a-5p	0.5651	miR-93-3p	0.3495
miR-93-3p	0.5561	let-7a-5p	0.3407
miR-27a	0.5409	miR-205-3p	0.3284
miR-27a-3p	0.5035	miR-1247-3p	0.2952
miR-1307-3p	0.4741	miR-3195	0.2885
miR-195-5p	0.4644	miR-26a-5p	0.2711
miR-15b-5p	0.4506	miR-191-5p	0.2387
miR-125a-3p	0.3816	let-7f-5p	0.2176
miR-1246	0.3763	miR-151a-5p	0.2102
miR-1247-3p	0.3627	miR-23b-3p	0.2093
miR-181a-3p	0.3601	miR-19a-5p	0.2073
miR-19b-3p	0.3384	miR-375-5p	0.1843
miR-877-5p	0.3353	miR-103a-3p	0.1817
miR-15a	0.3231	miR-4792	0.1764
miR-19a-3p	0.3143	miR-200a-5p	0.1657
miR-21	0.3099	miR-146a-5p	0.1560
miR-24-3p	0.3078	miR-27a-3p	0.1459
miR-4741	0.3008	miR-27a-5p	0.1443
miR-210-3p	0.2813	miR-1224-5p	0.1429
miR-224-5p	0.2761	miR-149-5p	0.1355
miR-4454	0.2729	miR-5100	0.1294
miR-20b	0.2704	let-7g-5p	0.1276
miR-149-3p	0.2558	miR-210-3p	0.1236
miR-30c-5p	0.2523	miR-222-3p	0.1140
miR-151a-5p	0.2460	miR-584-5p	0.1119
miR-92b-3p	0.2449	miR-17-5p	0.1114
let-7f-5p	0.2300	miR-30e-3p	0.1086
miR-375-5p	0.2274	miR-224-5p	0.1020
miR-1468-5p	0.2088	miR-150-3p	0.0969
miR-19a-5p	0.2026	miR-21	0.0961
miR-584-5p	0.1921	miR-4767	0.0945
miR-186-5p	0.1908	miR-7704	0.0939
miR-30b-5p	0.1890	miR-4741	0.0878
miR-1180-3p	0.1890	miR-222	0.0856
miR-222-3p	0.1886	miR-30c-5p	0.0852
miR-497-5p	0.1873	miR-132-3p	0.0834
miR-103a-3p	0.1817	miR-497-5p	0.0825
let-7g-5p	0.1764	miR-17-3p	0.0813
miR-30e-3p	0.1752	miR-574-3p	0.0748
miR-122-5p	0.1735	miR-93	0.0726
miR-146b-5p	0.1696	miR-4257	0.0723
miR-151a	0.1692	miR-30b-5p	0.0703
miR-23b-3p	0.1653	miR-30a-3p	0.0678
miR-196a-5p	0.1649	miR-675-3p	0.0646
miR-146a-5p	0.1634	miR-182-5p	0.0632
miR-203b-3p	0.1590	miR-574-5p	0.0619
miR-215-5p	0.1539	miR-186-5p	0.0597
miR-200a-5p	0.1397	miR-1307-3p	0.0579
miR-5100	0.1309	miR-215-5p	0.0571
miR-222	0.1262	miR-877-5p	0.0563
miR-182-5p	0.1262	miR-30d-5p	0.0561
miR-103b	0.1196	miR-99b-5p	0.0554
miR-149-5p	0.1174	miR-20b	0.0544
miR-195-3p	0.1103	miR-195-3p	0.0535
miR-185-5p	0.1081	miR-211	0.0512
miR-183-5p	0.1051	miR-1307-5p	0.0477
miR-30d-5p	0.1018	miR-224-3p	0.0465
miR-10b-5p	0.0943	miR-21-3p	0.0456
miR-103a-1-5p	0.0939	miR-375	0.0437
miR-205-3p	0.0939	miR-193b-3p	0.0397
miR-423-3p	0.0894	miR-15a	0.0392
miR-411-5p	0.0888	miR-10b-5p	0.0361
miR-21-3p	0.0852	miR-151a	0.0357
miR-130b-3p	0.0810	miR-17	0.0344
miR-222-5p	0.0804	miR-10a-3p	0.0331
miR-17-5p	0.0728	miR-196a-5p	0.0324
miR-34b-5p	0.0697	miR-185-5p	0.0317
miR-10a-3p	0.0682	miR-19a-3p	0.0307
miR-181a-5p	0.0582	miR-130a-3p	0.0288
miR-30b-3p	0.0571	miR-122-5p	0.0265
miR-142-5p	0.0570	miR-130a	0.0254
miR-152-3p	0.0563	miR-92b-3p	0.0250
miR-137	0.0561	miR-193a	0.0246
miR-221-5p	0.0549	miR-501	0.0243
miR-411	0.0512	miR-200b-3p	0.0237
miR-146b-3p	0.0437	miR-4772-3p	0.0235
miR-223-3p	0.0433	miR-34a-5p	0.0225
miR-193b-3p	0.0431	miR-126	0.0222
miR-34a-3p	0.0405	let-7b	0.0214
miR-193a-3p	0.0398	miR-149-3p	0.0201
miR-200b-3p	0.0393	miR-103a-1-5p	0.0199
let-7d-3p	0.0365	miR-221-5p	0.0199
miR-31-3p	0.0365	miR-675-5p	0.0198
miR-145-5p	0.0342	miR-211-5p	0.0194
miR-34b-3p	0.0310	miR-34b-5p	0.0192
miR-103a-2-5p	0.0300	miR-183-5p	0.0190
miR-10a	0.0279	miR-483-5p	0.0187
miR-193a	0.0247	miR-1468-5p	0.0183
miR-339-5p	0.0241	miR-152-3p	0.0176
miR-1260b	0.0241	miR-181a-5p	0.0170
miR-194-5p	0.0212	miR-203b-3p	0.0168
miR-205-5p	0.0210	miR-29b-3p	0.0165
miR-224-3p	0.0186	let-7d-3p	0.0164
miR-455-5p	0.0186	miR-191-3p	0.0159
miR-155-5p	0.0177	miR-15b-5p	0.0158
miR-34a-5p	0.0177	miR-31-3p	0.0152
miR-574-5p	0.0166	miR-423-3p	0.0137
miR-34c-5p	0.0162	miR-103b	0.0134
miR-577	0.0160	miR-1307	0.0133
miR-30a-3p	0.0153	miR-155	0.0129
miR-27a-5p	0.0142	miR-30e-5p	0.0129
miR-30c-1-3p	0.0139	miR-137	0.0126
miR-32-5p	0.0130	miR-455-5p	0.0124
miR-211	0.0130	miR-181b-5p	0.0123
miR-4772-3p	0.0129	miR-411-5p	0.0114
miR-211-5p	0.0127	miR-31-5p	0.0114
miR-452-5p	0.0126	miR-155-5p	0.0108
miR-203b-5p	0.0126	miR-20a	0.0106
miR-148a	0.0116	miR-1260b	0.0098
miR-93-5p	0.0107	miR-30c-2-3p	0.0097
miR-31-5p	0.0087	miR-23a-5p	0.0094
miR-1224-5p	0.0082	miR-222-5p	0.0092
miR-223	0.0077	miR-296-5p	0.0088
miR-18a	0.0075	miR-193a-3p	0.0087
miR-126	0.0073	miR-146b-5p	0.0085
let-7b	0.0071	miR-18a	0.0083
miR-221	0.0070	miR-10a	0.0081
miR-191-3p	0.0070	miR-205-5p	0.0078
miR-135b-5p	0.0070	miR-30b-3p	0.0077
miR-548c-5p	0.0069	miR-130a-5p	0.0076
miR-486-5p	0.0061	miR-103a-2-5p	0.0071
miR-96-5p	0.0057	miR-130b-3p	0.0069
miR-451a	0.0053	miR-577	0.0067
miR-200a-3p	0.0050	miR-361-5p	0.0066
miR-675-3p	0.0050	miR-411	0.0066
miR-181a-2-3p	0.0046	miR-34b-3p	0.0063
miR-93	0.0045	miR-194-5p	0.0060
miR-101-5p	0.0043	miR-32-5p	0.0058
miR-7-5p	0.0041	miR-9-5p	0.0058
miR-141-3p	0.0041	miR-150-5p	0.0057
miR-155	0.0036	miR-34c-5p	0.0056
miR-501	0.0035	miR-34a-3p	0.0055
miR-574-3p	0.0035	miR-339-5p	0.0054
miR-30e-5p	0.0031	miR-148a	0.0051
miR-125b-1-3p	0.0030	miR-4525	0.0049
miR-150-3p	0.0030	miR-181a-3p	0.0047
miR-34c-3p	0.0028	miR-20a-5p	0.0045
miR-142-3p	0.0028	miR-30c-1-3p	0.0044
miR-26a-5p	0.0026	miR-223-3p	0.0044
miR-203a-3p	0.0025	miR-29a	0.0044
miR-106b-3p	0.0024	miR-221	0.0042
miR-944	0.0023	miR-196a-3p	0.0036
miR-17-3p	0.0023	miR-452-5p	0.0035
miR-144-5p	0.0018	miR-135b-5p	0.0030
miR-429	0.0017	miR-142-5p	0.0029
miR-20a-5p	0.0016	miR-126-3p	0.0029
miR-135b	0.0015	miR-203b-5p	0.0028
miR-200c-3p	0.0014	miR-181a-2-3p	0.0025
miR-34a	0.0014	miR-7-5p	0.0024
miR-1236	0.0012	miR-200c-3p	0.0024
miR-449a	0.0011	miR-146b-3p	0.0022
miR-145	0.0008	miR-101-5p	0.0021
		miR-96-5p	0.0020
		miR-10b	0.0019
		miR-106b	0.0019
		miR-451a	0.0019
		miR-199a-3p	0.0019
		miR-548c-5p	0.0018
		miR-200a-3p	0.0017
		miR-34c-3p	0.0017
		miR-223	0.0016
		miR-141-5p	0.0014
		miR-142-3p	0.0014
		miR-203a-3p	0.0012
		miR-125b-1-3p	0.0010

**Table A4:** Complete miRNAs expression dataset for SC-TPA-EVs and IFP-MSC-TPA-EVs.

IFP-MSC Upregulated by TPA	IFP-MSC Downregulated by TPA	SC Upregulated by TPA	SC Downregulated by TPA
miR-451a	miR-486-5p	miR-30c-2-3p	miR-26a-5p
miR-1247-3p	miR-196a-3p	miR-181a-3p	miR-17-3p
miR-1246	miR-20a	miR-19b-3p	miR-150-3p
miR-877-5p	miR-501	miR-23a-5p	miR-574-3p
miR-96-5p	miR-4525	miR-1307-5p	miR-1224-5p
miR-193a-3p	miR-155	miR-15b-5p	miR-93
miR-1180-3p	miR-210-3p	miR-4767	miR-675-3p
miR-130a-3p	miR-106b	miR-149-3p	miR-27a-5p
miR-17-5p	miR-132-3p	miR-4257	miR-501
miR-34c-3p	miR-17	miR-130b-3p	miR-4454
miR-130a	miR-548c-5p	miR-1468-5p	miR-1246
miR-29b-3p	miR-9-5p	miR-7704	miR-30a-3p
miR-223-3p	miR-375	miR-19a-3p	miR-211
miR-142-3p	miR-675-5p	miR-146b-5p	miR-31-5p
miR-31-3p	miR-361-5p	miR-92b-3p	miR-574-5p
miR-199a-3p	miR-205-3p	miR-203b-3p	miR-205-3p
miR-24-3p	miR-142-5p	miR-223	miR-4772-3p
miR-186-5p	miR-211	miR-103b	miR-155
miR-34a-5p	miR-25-5p	miR-4792	miR-126
miR-126	miR-301a	miR-222-5p	miR-1180-3p
miR-200c-3p	miR-455-5p	miR-15a	miR-7-5p
miR-1290	miR-18a	miR-1307-3p	miR-224-3p
miR-222	miR-99a-5p	miR-411	miR-191-3p
miR-149-5p	miR-10b	miR-411-5p	miR-4644
miR-221-3p	miR-148a	miR-30b-3p	miR-455-5p
miR-101-5p	miR-483-5p	miR-34a-3p	miR-16-5p
miR-150-5p	miR-4306	miR-223-3p	miR-24-3p
miR-126-3p	miR-15a	miR-122-5p	miR-4306
miR-92b-3p	miR-215-5p	miR-423-3p	miR-34c-3p
miR-222-3p	miR-191-3p	miR-6126	miR-27a
miR-193a	miR-4767	miR-200b-3p	miR-211-5p
miR-195-5p	miR-93	miR-877-5p	miR-17-5p
miR-27a-3p	miR-4505	miR-3960	let-7b
miR-4454	miR-141	miR-183-5p	miR-221-3p
miR-99b-5p	miR-30c-5p	miR-196a-5p	miR-195-5p
miR-16-5p	miR-944	miR-3195	miR-192-5p
miR-194-5p	miR-224-5p	miR-20b	miR-20a-5p
miR-30b-3p	miR-145-5p	miR-34b-3p	miR-301a
miR-151a-5p	miR-130b-3p	miR-151a	miR-25-3p
miR-103a-3p	miR-10a-5p	miR-103a-1-5p	miR-34a-5p
miR-185-5p	miR-1307-5p	miR-4505	miR-23b-3p
miR-122-5p	miR-30d-5p	miR-193a-3p	miR-32-5p
miR-34b-3p	miR-150-3p	miR-203b-5p	miR-200a-5p
miR-10b-5p	miR-103b	miR-339-5p	miR-149-5p
miR-137	miR-452-5p	miR-137	miR-21-5p
miR-93-5p	miR-130a-5p	miR-103a-2-5p	miR-19a-5p
miR-26a-5p	miR-125b-1-3p	miR-34b-5p	
miR-25-3p	miR-4257	miR-194-5p	
miR-34b-5p	miR-191-5p	miR-99a-5p	
miR-577	miR-27a-5p	miR-10a	
miR-30e-3p	miR-93-3p	miR-451a	
miR-3960	miR-3195	miR-27a-3p	
miR-181b-5p	miR-16-2-3p	miR-4741	
miR-30c-1-3p	miR-675-3p	miR-181a-5p	
let-7a-5p	miR-4741	miR-185-5p	
miR-182-5p	miR-4488	miR-92a-3p	
miR-143	miR-181a-3p	miR-101-5p	
miR-23a-5p	miR-200b-3p	miR-21	
let-7g-5p	miR-339-5p	miR-152-3p	
miR-296-5p	miR-301a-3p	miR-186-5p	
miR-125b-2-3p	miR-34a-3p	miR-30c-1-3p	
miR-224-3p	miR-181a-2-3p	miR-4488	
miR-17-3p	miR-21-3p	miR-30c-5p	
miR-34c-5p	miR-135b-5p	miR-34c-5p	
let-7f-5p	miR-141-5p	miR-221-5p	
miR-423-3p	miR-497-5p	miR-224-5p	
miR-21-5p	miR-203b-3p	miR-205-5p	
miR-181a-5p	miR-574-5p	miR-215-5p	
miR-1468-5p	miR-101-3p	miR-30b-5p	
miR-30b-5p	miR-23a-3p	miR-452-5p	
miR-1236	miR-21	miR-96-5p	
miR-23a	miR-1307-3p	miR-10b-5p	
miR-211-5p	miR-149-3p	miR-10a-5p	
miR-103a-1-5p	miR-30a-3p	miR-16-2-3p	
miR-1307	miR-29a-3p	miR-1260b	
miR-23b-3p		miR-31-3p	
miR-195-3p		miR-191-5p	
miR-20b		miR-1290	
miR-4792		miR-210-3p	
miR-203b-5p		miR-497-5p	
miR-584-5p		let-7d-3p	
miR-10a		miR-301a-3p	
miR-411		miR-320b	
miR-5100		miR-195-3p	
miR-1260b		miR-10a-3p	
miR-15b-5p		miR-182-5p	
miR-29a		miR-101-3p	
let-7b		miR-142-3p	
miR-411-5p		miR-21-3p	
miR-4772-3p		miR-181a-2-3p	
miR-27a		miR-30d-5p	
miR-222-5p		let-7c-5p	
let-7c-5p		miR-584-5p	
miR-7-5p		miR-125b-5p	
miR-205-5p		miR-221	
miR-1224-5p		miR-222-3p	
miR-23b-5p		miR-155-5p	
miR-574-3p		miR-30e-3p	
miR-200a-5p		miR-93-3p	
		miR-18a	
		miR-222	
		miR-135b-5p	
		miR-148a	
		miR-30e-5p	
		let-7g-5p	
		miR-125b-2-3p	
		miR-375-5p	
		miR-1247-3p	
		miR-548c-5p	
		miR-151a-5p	
		miR-23a-3p	
		miR-23a	
		miR-125a-3p	
		miR-577	
		miR-193b-3p	
		let-7f-5p	
		miR-146a-5p	
		miR-5100	
		miR-193a	
		miR-103a-3p	

## References

[1] Damerell V., Pepper M.S., Prince S. (2021). Molecular mechanisms underpinning sarcomas and implications for current and future therapy. Signal Transduct. Target. Ther..

[2] Kouroupis D., Sanjurjo-Rodriguez C., Jones E., Correa D. (2019). Mesenchymal Stem Cell Functionalization for Enhanced Therapeutic Applications. Tissue Eng. Part B Rev..

[3] Jones E.A., Giannoudis P.V., Kouroupis D. (2016). Bone repair with skeletal stem cells: Rationale, progress to date and clinical application. Ther. Adv. Musculoskelet. Dis..

[4] Caplan A.I., Correa D. (2011). The MSC: An injury drugstore. Cell Stem Cell.

[5] Scioli M.G., Terriaca S., Fiorelli E., Storti G., Fabbri G., Cervelli V., Orlandi A. (2021). Extracellular Vesicles and Cancer Stem Cells in Tumor Progression: New Therapeutic Perspectives. Int. J. Mol. Sci..

[6] Nikfarjam S., Rezaie J., Zolbanin N.M., Jafari R. (2020). Mesenchymal stem cell derived-exosomes: A modern approach in translational medicine. J. Transl. Med..

[7] Galoian K., Temple T.H., Galoyan A. (2011). Cytostatic effect of the hypothalamic cytokine PRP-1 is mediated by mTOR and cMyc inhibition in high grade chondrosarcoma. Neurochem. Res..

[8] Galoian K., Bilbao D., Denny C., Campos Gallego N., Roberts E., Martinez D., Temple H.T. (2024). Targeting cancer stem cells by TPA leads to inhibition of refractory sarcoma and extended overall survival. Mol. Ther. Oncol..

[9] Galoyan A. (2000). Neurochemistry of brain neuroendocrine immune system: Signal molecules. Neurochem. Res..

[10] Galoian K., Luo S., Qureshi A., Patel P., Price R., Morse A.S., Chailyan G., Abrahamyan S., Temple H.T. (2016). Effect of cytostatic proline rich polypeptide-1 on tumor suppressors of inflammation pathway signaling in chondrosarcoma. Mol. Clin. Oncol..

[11] Galoian K.A., Guettouche T., Issac B., Qureshi A., Temple H.T. (2014). Regulation of onco and tumor suppressor MiRNAs by mTORC1 inhibitor PRP-1 in human chondrosarcoma. Tumour Biol..

[12] Moran A., Hoyt A., Sedani A., Granger C., Saigh S., Blonska M., Zhao-Ju L., Conway S.A., Pretell J., Brown J. (2020). Proline-rich polypeptide-1 decreases cancer stem cell population by targeting BAFF chromatin-remodeling complexes in human chondrosarcoma JJ012 cells. Oncol. Rep..

[13] Galoian K., Qureshi A., D’Ippolito G., Schiller P.C., Molinari M., Johnstone A.L., Brothers S.P., Paz A.C., Temple H.T. (2015). Epigenetic regulation of embryonic stem cell marker miR302C in human chondrosarcoma as determinant of antiproliferative activity of proline-rich polypeptide 1. Int. J. Oncol..

[14] Yenkoyan K., Safaryan K., Chavushyan V., Meliksetyan I., Navasardyan G., Sarkissian J., Galoyan A., Aghajanov M. (2011). Neuroprotective action of proline-rich polypeptide-1 in beta-amyloid induced neurodegeneration in rats. Brain Res. Bull..

[15] Chailakhyan R.K., Gerasimov Y.V., Chailakhyan M.R., Galoyan A.A. (2010). Proline-rich hypothalamic polypeptide has opposite effects on the proliferation of human normal bone marrow stromal cells and human giant-cell tumour stromal cells. Neurochem. Res..

[16] Galoyan A.A., Sarkissian J.S., Kipriyan T.K., Sarkissian E.J., Chavushyan E.A., Sulkhanyan R.M., Meliksetyan I.B., Abrahamyan S.S., Grigorian Y., Avetisyan Z.A. (2001). Protective effect of a new hypothalamic peptide against cobra venom and trauma-induced neuronal injury. Neurochem. Res..

[17] Granger C.J., Hoyt A.K., Moran A., Becker B., Sedani A., Saigh S., Conway S.A., Brown J., Galoian K. (2020). Cancer stem cells as a therapeutic target in 3D tumor models of human chondrosarcoma: An encouraging future for proline rich polypeptide-1. Mol. Med. Rep..

[18] Kalluri R., LeBleu V.S. (2020). The biology, function, and biomedical applications of exosomes. Science.

[19] Garcia-Aponte O.F., Kahlenberg S., Kouroupis D., Egger D., Kasper C. (2025). Effects of Hydrogels on Mesenchymal Stem/Stromal Cells Paracrine Activity and Extracellular Vesicles Production. J. Extracell. Vesicles.

[20] Kouroupis D., Kaplan L.D., Ricordi C., Best T.M. (2023). Mesenchymal Stem/Stromal Cell-Derived Small Extracellular Vesicles (MSC-sEVs): A Promising Treatment Modality for Diabetic Foot Ulcer. Bioengineering.

[21] Verma N., Arora S., Singh A.K., Kumar A. (2025). Extracellular Vesicle-Associated miRNAs in Cornea Health and Disease: Diagnostic Potential and Therapeutic Implications. Targets.

[22] Greening D.W., Xu R., Rai A., Suwakulsiri W., Chen M., Simpson R.J. (2025). Clinical relevance of extracellular vesicles in cancer—Therapeutic and diagnostic potential. Nat. Rev. Clin. Oncol..

[23] Kuang L., Wu L., Li Y. (2025). Extracellular vesicles in tumor immunity: Mechanisms and novel insights. Mol. Cancer.

[24] Goto K., Watanabe D., Yanagida K., Takagi T., Mizushima A. (2025). Harnessing miRNA-Containing Extracellular Vesicles from Mesenchymal Stromal Cell-Derived Extracellular Vesicles for Regeneration of Bone Defects: A Narrative Review of Mechanisms, Biomaterials, and Clinical Translation. Cancers.

[25] Nelson H., Qu S., Franklin J.L., Liu Q., Pua H.H., Vickers K.C., Weaver A.M., Coffey R.J., Patton J.G. (2024). Extracellular RNA in oncogenesis, metastasis and drug resistance. RNA Biol..

[26] Kim Y., Kim J.Y., Moon S., Lee H., Lee S., Kim J.Y., Kim M.W., Kim S.I. (2024). Tumor-derived EV miRNA signatures surpass total EV miRNA in supplementing mammography for precision breast cancer diagnosis. Theranostics.

[27] Kou M., Huang L., Yang J., Chiang Z., Chen S., Liu J., Guo L., Zhang X., Zhou X., Xu X. (2022). Mesenchymal stem cell-derived extracellular vesicles for immunomodulation and regeneration: A next generation therapeutic tool?. Cell Death Dis..

[28] Lamichhane A., Tavana H. (2024). Three-Dimensional Tumor Models to Study Cancer Stemness-Mediated Drug Resistance. Cell Mol. Bioeng..

[29] Jovanovic Stojanov S., Grozdanic M., Ljujic M., Dragicevic S., Dragoj M., Dinic J. (2025). Cancer 3D Models: Essential Tools for Understanding and Overcoming Drug Resistance. Oncol. Res..

[30] Greif D.N., Kouroupis D., Murdock C.J., Griswold A.J., Kaplan L.D., Best T.M., Correa D. (2020). Infrapatellar Fat Pad/Synovium Complex in Early-Stage Knee Osteoarthritis: Potential New Target and Source of Therapeutic Mesenchymal Stem/Stromal Cells. Front. Bioeng. Biotechnol..

[31] Philippon M., Labib R., Ley M., Kaplan L.D., Mendez A.J., Best T.M., Kouroupis D. (2025). Characterization of Extracellular Vesicles from Infrapatellar Fat Pad Mesenchymal Stem/Stromal Cells Expanded Using Regulatory-Compliant Media and Inflammatory/Hormonal Priming. Cells.

[32] Kouroupis D., Kaplan L.D., Best T.M. (2022). Human infrapatellar fat pad mesenchymal stem cells show immunomodulatory exosomal signatures. Sci. Rep..

[33] Kouroupis D., Bowles A.C., Willman M.A., Perucca Orfei C., Colombini A., Best T.M., Kaplan L.D., Correa D. (2019). Infrapatellar fat pad-derived MSC response to inflammation and fibrosis induces an immunomodulatory phenotype involving CD10-mediated Substance P degradation. Sci. Rep..

[34] Kouroupis D., Kaplan L.D., Huard J., Best T.M. (2023). CD10-Bound Human Mesenchymal Stem/Stromal Cell-Derived Small Extracellular Vesicles Possess Immunomodulatory Cargo and Maintain Cartilage Homeostasis under Inflammatory Conditions. Cells.

[35] Fan Y., Siklenka K., Arora S.K., Ribeiro P., Kimmins S., Xia J. (2016). miRNet—Dissecting miRNA-target interactions and functional associations through network-based visual analysis. Nucleic Acids Res..

[36] Chen Y., Wang X. (2020). miRDB: An online database for prediction of functional microRNA targets. Nucleic Acids Res..

[37] Yan C., Zhou Q.Y., Wu J., Xu N., Du Y., Li J., Liu J.X., Koda S., Zhang B.B., Yu Q. (2021). Csi-let-7a-5p delivered by extracellular vesicles from a liver fluke activates M1-like macrophages and exacerbates biliary injuries. Proc. Natl. Acad. Sci. USA.

[38] Sun L.L., Li W.D., Lei F.R., Li X.Q. (2018). The regulatory role of microRNAs in angiogenesis-related diseases. J. Cell Mol. Med..

[39] Chen Y., Gorski D.H. (2008). Regulation of angiogenesis through a microRNA (miR-130a) that down-regulates antiangiogenic homeobox genes GAX and HOXA5. Blood.

[40] Chen Z., Qin Y. (2025). Role of miRNA-145-5p in cancer (Review). Oncol. Rep..

[41] Sabouni E., Nejad M.M., Mojtabavi S., Khoshduz S., Mojtabavi M., Nadafzadeh N., Nikpanjeh N., Mirzaei S., Hashemi M., Aref A.R. (2023). Unraveling the function of epithelial-mesenchymal transition (EMT) in colorectal cancer: Metastasis, therapy response, and revisiting molecular pathways. Biomed. Pharmacother..

[42] Hussen B.M., Abdullah S.R., Rasul M.F., Jawhar Z.H., Faraj G.S.H., Kiani A., Taheri M. (2023). MiRNA-93: A novel signature in human disorders and drug resistance. Cell Commun. Signal.

[43] Su Z., Jiang G., Chen J., Liu X., Zhao H., Fang Z., He Y., Jiang X., Xu G. (2020). MicroRNA-429 inhibits cancer cell proliferation and migration by targeting AKT1 in renal cell carcinoma. Mol. Clin. Oncol..

[44] Liang L., Zhao L., Zan Y., Zhu Q., Ren J., Zhao X. (2017). MiR-93-5p enhances growth and angiogenesis capacity of HUVECs by down-regulating EPLIN. Oncotarget.

[45] Li J., Du L., Yang Y., Wang C., Liu H., Wang L., Zhang X., Li W., Zheng G., Dong Z. (2013). MiR-429 is an independent prognostic factor in colorectal cancer and exerts its anti-apoptotic function by targeting SOX2. Cancer Lett..

[46] Wang F.X., Mu G., Yu Z.H., Qin Z.S., Zhao X., Shi Z.A., Fan X., Liu L., Chen Y., Zhou J. (2025). MiR-451 in Inflammatory Diseases: Molecular Mechanisms, Biomarkers, and Therapeutic Applications-A Comprehensive Review Beyond Oncology. Curr. Issues Mol. Biol..

[47] Liu J.S., Du J., Cheng X., Zhang X.Z., Li Y., Chen X.L. (2019). Exosomal miR-451 from human umbilical cord mesenchymal stem cells attenuates burn-induced acute lung injury. J. Chin. Med. Assoc..

[48] Khosravi M., Azarpira N., Shamdani S., Hojjat-Assari S., Naserian S., Karimi M.H. (2018). Differentiation of umbilical cord derived mesenchymal stem cells to hepatocyte cells by transfection of miR-106a, miR-574-3p, and miR-451. Gene.

[49] Yu T., Chen D., Zhang L., Wan D. (2019). microRNA-26a-5p Promotes Proliferation and Migration of Osteosarcoma Cells by Targeting HOXA5 in vitro and in vivo. Onco Targets Ther..

[50] Mohd Yacob A., Muhamad N.A., Chang K.M., Akmal Hisham H., Mat Yusoff Y., Ibrahim L. (2022). Hsa-miR-181a-5p, hsa-miR-182-5p, and hsa-miR-26a-5p as potential biomarkers for BCR-ABL1 among adult chronic myeloid leukemia treated with tyrosine kinase inhibitors at the molecular response. BMC Cancer.

[51] Minami Y., Kohsaka S., Tsuda M., Yachi K., Hatori N., Tanino M., Kimura T., Nishihara H., Minami A., Iwasaki N. (2014). SS18-SSX-regulated miR-17 promotes tumor growth of synovial sarcoma by inhibiting p21WAF1/CIP1. Cancer Sci..

[52] Wan Y., Cui R., Gu J., Zhang X., Xiang X., Liu C., Qu K., Lin T. (2017). Identification of Four Oxidative Stress-Responsive MicroRNAs, miR-34a-5p, miR-1915-3p, miR-638, and miR-150-3p, in Hepatocellular Carcinoma. Oxid. Med. Cell Longev..

[53] Ameri A., Ahmed H.M., Pecho R.D.C., Arabnozari H., Sarabadani H., Esbati R., Mirabdali S., Yazdani O. (2023). Diverse activity of miR-150 in Tumor development: Shedding light on the potential mechanisms. Cancer Cell Int..

[54] Xu H., Liu X., Zhou J., Chen X., Zhao J. (2016). miR-574-3p acts as a tumor promoter in osteosarcoma by targeting SMAD4 signaling pathway. Oncol. Lett..

[55] Chu D., Liu T., Yao Y., Luan N. (2021). LINC00997/MicroRNA 574-3p/CUL2 Promotes Cervical Cancer Development via Mitogen-Activated Protein Kinase Signaling. Mol. Cell Biol..

[56] Ma M., Li J., Zhang Z., Sun J., Liu Z., Zeng Z., Ouyang S., Kang W. (2022). The Role and Mechanism of microRNA-1224 in Human Cancer. Front. Oncol..

[57] Fang L., Deng Z., Shatseva T., Yang J., Peng C., Du W.W., Yee A.J., Ang L.C., He C., Shan S.W. (2011). MicroRNA miR-93 promotes tumor growth and angiogenesis by targeting integrin-beta8. Oncogene.

[58] Greither T., Vorwerk F., Kappler M., Bache M., Taubert H., Kuhnt T., Hey J., Eckert A.W. (2017). Salivary miR-93 and miR-200a as post-radiotherapy biomarkers in head and neck squamous cell carcinoma. Oncol. Rep..

[59] Zhang H., Zhang J., Meng F., Zhu H., Yan H., Guo Y., Zhang S. (2019). MicroRNA-93 promotes the tumorigenesis of osteosarcoma by targeting TIMP2. Biosci. Rep..

[60] Wang F., Rong L., Zhang Z., Li M., Ma L., Ma Y., Xie X., Tian X., Yang Y. (2020). LncRNA H19-Derived miR-675-3p Promotes Epithelial-Mesenchymal Transition and Stemness in Human Pancreatic Cancer Cells by targeting the STAT3 Pathway. J. Cancer.

[61] Li P., Luo X., Xie Y., Li P., Hu F., Chu J., Chen X., Song W., Wang A., Tian G. (2020). GC-Derived EVs Enriched with MicroRNA-675-3p Contribute to the MAPK/PD-L1-Mediated Tumor Immune Escape by Targeting CXXC4. Mol. Ther. Nucleic Acids.

[62] Salah Z., Arafeh R., Maximov V., Galasso M., Khawaled S., Abou-Sharieha S., Volinia S., Jones K.B., Croce C.M., Aqeilan R.I. (2015). miR-27a and miR-27a* contribute to metastatic properties of osteosarcoma cells. Oncotarget.

[63] Tang C., Li C., Chen C., Chen T., Zhu J., Sun M., Wang P., Han C. (2024). LINC01234 promoted malignant behaviors of breast cancer cells via hsa-miR-30c-2-3p/CCT4/mTOR signaling pathway. Taiwan J. Obstet. Gynecol..

[64] Huang X., Jia Y., Shi H., Fan H., Sun L., Zhang H., Wang Y., Chen J., Han J., Wang M. (2023). miR-30c-2-3p suppresses the proliferation of human renal cell carcinoma cells by targeting TOP2A. Asian Biomed. (Res. Rev. News).

[65] Mitsueda R., Toda H., Shinden Y., Fukuda K., Yasudome R., Kato M., Kikkawa N., Ohtsuka T., Nakajo A., Seki N. (2023). Oncogenic Targets Regulated by Tumor-Suppressive miR-30c-1-3p and miR-30c-2-3p: TRIP13 Facilitates Cancer Cell Aggressiveness in Breast Cancer. Cancers.

[66] Liu B., Lu K., Yuan L., Li X., Lan L., Han S. (2024). Hsa-miR-181a-2-3p inhibits the oncogenicity of colon cancer by directly targeting STING. Aging.

[67] McIntyre G., Jackson Z., Colina J., Sekhar S., DiFeo A. (2024). miR-181a: Regulatory roles, cancer-associated signaling pathway disruptions, and therapeutic potential. Expert Opin. Ther. Targets.

[68] Duca R.B., Massillo C., Dalton G.N., Farre P.L., Grana K.D., Gardner K., De Siervi A. (2021). MiR-19b-3p and miR-101-3p as potential biomarkers for prostate cancer diagnosis and prognosis. Am. J. Cancer Res..

[69] He Y., Meng C., Shao Z., Wang H., Yang S. (2014). MiR-23a functions as a tumor suppressor in osteosarcoma. Cell Physiol. Biochem..

[70] Wang N., Tan H.Y., Feng Y.G., Zhang C., Chen F., Feng Y. (2018). microRNA-23a in Human Cancer: Its Roles, Mechanisms and Therapeutic Relevance. Cancers.

[71] Huang W., Zhang C., Xiong S., Zhou X., Wang G., Guo J. (2022). miR-1307-5p suppresses proliferation and tumorigenesis of bladder cancer via targeting MDM4 and the Hippo signaling pathway. Discov. Oncol..

[72] Liu H.Q., Shu X., Ma Q., Wang R., Huang M.Y., Gao X., Liu Y.N. (2020). Identifying specific miRNAs and associated mRNAs in CD44 and CD90 cancer stem cell subtypes in gastric cancer cell line SNU-5. Int. J. Clin. Exp. Pathol..

[73] Weng Y., Shen Y., He Y., Pan X., Xu J., Jiang Y., Zhang Q., Wang S., Kong F., Zhao S. (2018). The miR-15b-5p/PDK4 axis regulates osteosarcoma proliferation through modulation of the Warburg effect. Biochem. Biophys. Res. Commun..

[74] Teng W., Qiu C., He Z., Wang G., Xue Y., Hui X. (2017). Linc00152 suppresses apoptosis and promotes migration by sponging miR-4767 in vascular endothelial cells. Oncotarget.

[75] Yang D., Du G., Xu A., Xi X., Li D. (2017). Expression of miR-149-3p inhibits proliferation, migration, and invasion of bladder cancer by targeting S100A4. Am. J. Cancer Res..

[76] Wang Y., Song Y., Liu Z., Li J., Wang G., Pan H., Zheng Z. (2023). miR-149-3p suppresses the proliferation and metastasis of glioma cells by targeting the CBX2/Wnt/beta-catenin pathway. Exp. Ther. Med..

[77] Kataoka S., Arita T., Konishi H., Yamamoto Y., Shibata R., Yamamoto T., Shibamoto J., Furuke H., Takabatake K., Takaki W. (2022). The Role of Inflammation-associated microRNA-4257 as a Promoter of Malignancy in Colorectal Cancer. Anticancer. Res..

[78] Huang S., Xue P., Han X., Zhang C., Yang L., Liu L., Wang X., Li H., Fu J., Zhou Y. (2020). Exosomal miR-130b-3p targets SIK1 to inhibit medulloblastoma tumorigenesis. Cell Death Dis..

[79] Yang Z., Tang Y., Wu X., Wang J., Yao W. (2025). MicroRNA-130b Suppresses Malignant Behaviours and Inhibits the Activation of the PI3K/Akt Signaling Pathway by Targeting MET in Pancreatic Cancer. Biochem. Genet..

[80] Jiang K., Zhi T., Xu W., Xu X., Wu W., Yu T., Nie E., Zhou X., Bao Z., Jin X. (2017). MicroRNA-1468-5p inhibits glioma cell proliferation and induces cell cycle arrest by targeting RRM1. Am. J. Cancer Res..

[81] Mahlab-Aviv S., Zohar K., Cohen Y., Peretz A.R., Eliyahu T., Linial M., Sperling R. (2020). Spliceosome-Associated microRNAs Signify Breast Cancer Cells and Portray Potential Novel Nuclear Targets. Int. J. Mol. Sci..

[82] Polini B., Carpi S., Doccini S., Citi V., Martelli A., Feola S., Santorelli F.M., Cerullo V., Romanini A., Nieri P. (2020). Tumor Suppressor Role of hsa-miR-193a-3p and -5p in Cutaneous Melanoma. Int. J. Mol. Sci..

[83] Peng T., Yang F., Sun Z., Yan J. (2022). miR-19a-3p Facilitates Lung Adenocarcinoma Cell Phenotypes by Inhibiting TEK. Cancer Biother. Radiopharm..

[84] Pisano A., Grinan-Lison C., Farace C., Fiorito G., Fenu G., Jimenez G., Scognamillo F., Pena-Martin J., Naccarati A., Proll J. (2020). The Inhibitory Role of miR-486-5p on CSC Phenotype Has Diagnostic and Prognostic Potential in Colorectal Cancer. Cancers.

[85] Etzi F., Grinan-Lison C., Fenu G., Gonzalez-Titos A., Pisano A., Farace C., Sabalic A., Picon-Ruiz M., Marchal J.A., Madeddu R. (2024). The Role of miR-486-5p on CSCs Phenotypes in Colorectal Cancer. Cancers.

[86] Khalilian S., Hosseini Imani S.Z., Abedinlou H., Omrani M.A., Ghafouri-Fard S. (2024). miR-196a in the carcinogenesis and other disorders with an especial focus on its biomarker capacity. Pathol. Res. Pract..

[87] Chen Y., Huang S., Wu B., Fang J., Zhu M., Sun L., Zhang L., Zhang Y., Sun M., Guo L. (2017). Transforming growth factor-beta1 promotes breast cancer metastasis by downregulating miR-196a-3p expression. Oncotarget.

[88] Milevskiy M.J.G., Gujral U., Del Lama Marques C., Stone A., Northwood K., Burke L.J., Gee J.M.W., Nephew K., Clark S., Brown M.A. (2019). MicroRNA-196a is regulated by ER and is a prognostic biomarker in ER+ breast cancer. Br. J. Cancer.

[89] Dioguardi M., Cantore S., Sovereto D., La Femina L., Caloro G.A., Spirito F., Scacco S., Di Cosola M., Lo Muzio L., Troiano G. (2022). Potential Role of miR-196a and miR-196b as Prognostic Biomarkers of Survival in Head and Neck Squamous Cell Carcinoma: A Systematic Review, Meta-Analysis and Trial Sequential Analysis. Life.

[90] Wang Y.X., Peng Z.L., Sun Z.W., Pan Y.J., Ai H., Mai Z.H. (2023). MiR-20a promotes osteogenic differentiation in bone marrow-derived mesenchymal stem/stromal cells and bone repair of the maxillary sinus defect model in rabbits. Front. Bioeng. Biotechnol..

[91] Xu K., Han B., Bai Y., Ma X.Y., Ji Z.N., Xiong Y., Miao S.K., Zhang Y.Y., Zhou L.M. (2019). MiR-451a suppressing BAP31 can inhibit proliferation and increase apoptosis through inducing ER stress in colorectal cancer. Cell Death Dis..

[92] Azizi M., Andalib A., Rezaei M. (2025). Investigating the potential role of miR-451a as a tumor suppressor in immunogenic cell death induction and dendritic cell maturation in colorectal cancer cell lines. Cancer Cell Int..

[93] Tan J., Li C., Ren L., Zhu X., Hua F., Fu Y. (2022). miR-451a suppresses papillary thyroid cancer cell proliferation and invasion and facilitates apoptosis through targeting DCBLD2 and AKT1. Mol. Cell Probes.

[94] Chen J., Zhang X., Zhang G., Zhu F., Liu W. (2025). Serum-derived exosomal miR-7977 combined with miR-451a as a potential biomarker for pancreatic ductal adenocarcinoma. BMC Cancer.

[95] Wang F.X., Shi Z.A., Mu G. (2024). Regulation of immune cells by miR-451 and its potential as a biomarker in immune-related disorders: A mini review. Front. Immunol..

[96] Youness R.A., Elemam N.M., Abdelhamid A.M., Mohamed A.H., Elsherbiny L.M., Ramzy A., Assal R.A. (2025). Macrophage migration inhibitory factor (MIF) and the tumor ecosystem: A tale of inflammation, immune escape, and tumor growth. Front. Immunol..

[97] Lukomska A., Theune W.C., Frost M.P., Xing J., Kearney A., Trakhtenberg E.F. (2024). Upregulation of developmentally-downregulated miR-1247-5p promotes neuroprotection and axon regeneration in vivo. Neurosci. Lett..

[98] Huynh K.Q., Le A.T., Phan T.T., Ho T.T., Pho S.P., Nguyen H.T., Le B.T., Nguyen T.T., Nguyen S.T. (2023). The Diagnostic Power of Circulating miR-1246 in Screening Cancer: An Updated Meta-analysis. Oxid. Med. Cell Longev..

[99] Zhong L., Wang J., Wang P., Liu X., Liu P., Cheng X., Cao L., Wu H., Chen J., Zhou L. (2023). Neural stem cell-derived exosomes and regeneration: Cell-free therapeutic strategies for traumatic brain injury. Stem Cell Res. Ther..

[100] Shen Y., Zhang Y., Wang Q., Jiang B., Jiang X., Luo B. (2024). MicroRNA-877-5p promotes osteoblast differentiation by targeting EIF4G2 expression. J. Orthop. Surg. Res..

[101] Rahimi H.R., Mojarrad M., Moghbeli M. (2022). MicroRNA-96: A therapeutic and diagnostic tumor marker. Iran. J. Basic Med. Sci..

[102] Hong Y., Liang H., Uzair Ur R., Wang Y., Zhang W., Zhou Y., Chen S., Yu M., Cui S., Liu M. (2016). miR-96 promotes cell proliferation, migration and invasion by targeting PTPN9 in breast cancer. Sci. Rep..

[103] Zajac A.E., Czarnecka A.M., Rutkowski P. (2023). The Role of Macrophages in Sarcoma Tumor Microenvironment and Treatment. Cancers.

[104] Baron M., Drohat P., Crawford B., Hornicek F.J., Best T.M., Kouroupis D. (2023). Mesenchymal Stem/Stromal Cells: Immunomodulatory and Bone Regeneration Potential after Tumor Excision in Osteosarcoma Patients. Bioengineering.

[105] Reed T., Schorey J., D’Souza-Schorey C. (2021). Tumor-Derived Extracellular Vesicles: A Means of Co-opting Macrophage Polarization in the Tumor Microenvironment. Front. Cell Dev. Biol..

[106] Welsh J.A., Goberdhan D.C.I., O’Driscoll L., Buzas E.I., Blenkiron C., Bussolati B., Cai H., Di Vizio D., Driedonks T.A.P., Erdbrugger U. (2024). Minimal information for studies of extracellular vesicles (MISEV2023): From basic to advanced approaches. J. Extracell. Vesicles.

[107] Mizenko R.R., Feaver M., Bozkurt B.T., Lowe N., Nguyen B., Huang K.W., Wang A., Carney R.P. (2024). A critical systematic review of extracellular vesicle clinical trials. J. Extracell. Vesicles.

[108] Rezaie J., Feghhi M., Etemadi T. (2022). A review on exosomes application in clinical trials: Perspective, questions, and challenges. Cell Commun. Signal.

[109] Witwer K.W., Thery C. (2019). Extracellular vesicles or exosomes? On primacy, precision, and popularity influencing a choice of nomenclature. J. Extracell. Vesicles.

[110] Jia Y., Yu L., Ma T., Xu W., Qian H., Sun Y., Shi H. (2022). Small extracellular vesicles isolation and separation: Current techniques, pending questions and clinical applications. Theranostics.

[111] Burnouf T., Agrahari V., Agrahari V. (2019). Extracellular Vesicles As Nanomedicine: Hopes And Hurdles In Clinical Translation. Int. J. Nanomed..

[112] Su X., Wang H., Li Q., Chen Z. (2025). Extracellular Vesicles: A Review of Their Therapeutic Potentials, Sources, Biodistribution, and Administration Routes. Int. J. Nanomed..

[113] Drohat P., Baron M., Kaplan L.D., Best T.M., Kouroupis D. (2025). Long-Acting Extracellular Vesicle-Based Biologics in Osteoarthritis Immunotherapy. Bioengineering.

[114] O’Brien K., Ughetto S., Mahjoum S., Nair A.V., Breakefield X.O. (2022). Uptake, functionality, and re-release of extracellular vesicle-encapsulated cargo. Cell Rep..

